# Biomimetic Marine-Sponge-Derived Spicule-Microparticle-Mediated Biomineralization and YAP/TAZ Pathway for Bone Regeneration In Vivo

**DOI:** 10.34133/bmr.0056

**Published:** 2024-07-25

**Authors:** Sumi Choi, Jung Hun Kim, Tae Hoon Kang, Young-Hyeon An, Sang Jin Lee, Nathaniel S. Hwang, Su-Hwan Kim

**Affiliations:** ^1^ Department of Chemical Engineering (BK21 FOUR), Dong-A University, Busan 49315, Republic of Korea.; ^2^ School of Chemical and Biological Engineering, Seoul National University, Seoul 08826, Republic of Korea.; ^3^ Interdisciplinary Program in Bioengineering, Seoul National University, Seoul 08826, Republic of Korea.; ^4^ Bio-MAX/N-Bio, Seoul National University, Seoul 08826, Republic of Korea.; ^5^ Biofunctional Materials, Division of Applied Oral Sciences and Community Dental Care, Faculty of Dentistry, The University of Hong Kong, Sai Ying Pun, Hong Kong Special Administrative Region.

## Abstract

Marine-sponge-derived spicule microparticles (SPMs) possess unique structural and compositional features suitable for bone tissue engineering. However, significant challenges remain in establishing their osteogenic mechanism and practical application in animal models. This study explores the biomimetic potential of SPM in orchestrating biomineralization behavior and modulating the Yes-associated protein 1/transcriptional coactivator with PDZ-binding motif (YAP/TAZ) pathway both in vitro and in vivo. Characterization of SPM revealed a structure comprising amorphous silica oxide mixed with collagen and trace amounts of calcium and phosphate ions, which have the potential to facilitate biomineralization. Structural analysis indicated dynamic biomineralization from SPM to hydroxyapatite, contributing to both in vitro and in vivo osteoconductions. In vitro assessment demonstrated dose-dependent increases in osteogenic gene expression and bone morphogenetic protein-2 protein in response to SPM. In addition, focal adhesion mediated by silica diatoms induced cell spreading on the surface of SPM, leading to cell alignment in the direction of SPM. Mechanical signals from SPM subsequently increased the expression of YAP/TAZ, thereby inducing osteogenic mechanotransduction. The osteogenic activity of SPM-reinforced injectable hydrogel was evaluated in a mouse calvaria defect model, demonstrating rapid vascularized bone regeneration. These findings suggest that biomimetic SPM holds significant promise for regenerating bone tissue.

## Introduction

The bone defects are typically caused by degenerative disease, cancerous bone tumors, traumatic injury, and unexpected traffic accidents [1]. Since bone tissues are insufficient to regenerate large damages, clinical interventions are required to restore the structure and functions of the bone [2]. The current gold standard in treatments of bone fractures is bone autografts [3], the second most common transplanted tissue in the United States and available in cortical, bone marrow aspirate, and cancellous forms. However, these are limited to correcting irregular bone defects due to a shortage of tissue donors, incompatibility, and side effects such as immune response. Alternatively, bone xenografts from nonhuman species are alternative options to compensate for weaknesses in autografts. Although antigenicity, the abundance of donors, and its competitive price are attractive advantages in xenografts [4], the potential risks of functional incompatibility due to genetic and physiological differences (e.g., size, anatomical structure, and vascular architecture changes) are an inherent challenge to preventing commercialization in routine usage for orthopedic transplantation [5].

Recently, alternative strategies have been considered using bioceramics or biomimetic inorganic particles as appropriate solutions to promote new bone regeneration. These have been utilized in bone tissue engineering to recapitulate bone’s physical structures and mineral components [6,7]. Inorganic particles including bioglass, hydroxyapatite (HAP), and β-tricalcium phosphate promote osteogenic differentiation of stem cells and bone formation during bone remodeling [8]. The proposed osteogenic mechanism of such inorganic particles has relied on inorganic transformation to a hydroxycarbonate-rich layer that resembles HAPs in bone tissue [9]. In biological fluids, osteoconductive ions such as calcium, magnesium, or silica ions are released from inorganic particles, and hydroxycarbonate-rich layers are formed on the surface of inorganic particles, which promote chemical bonding to native bone tissue and stimulate endogenous cells to form bone tissue networks [10]. However, the current use of synthetic substitutes is accompanied by acknowledged limitations, encompassing issues of biocompatibility, mechanical properties, degradation, and the challenge of replicating the intricate biochemical signaling present in native tissues [11,12]. Consequently, there is a burgeoning interest in naturally derived substances for bone regeneration, as these materials offer the potential to overcome the noted drawbacks and align more closely with the biological and mechanical requirements of native bone tissue [13].

Remarkably, marine organisms have interesting structures and chemical compositions are potential resources of bone tissue engineering for the development of translational medical devices [14,15]. Among them, marine sponges are a new class of tissue engineering materials that recapitulate the structure and chemical properties of bone tissue [16]. The fundamental structure of marine sponges is the inorganic skeleton surrounding the fibrillar-collagen-based extracellular matrix. The skeleton called a spicule is formed of amorphous, noncrystalline silica dioxide (SiO_2_) in Demospongiae [17]. The spicule is enzymatically synthesized by silicatein and has distinguished properties such as flexibility, stability, and less fragility than silica bioglass due to its hydrated and layered structure. Since silica ions can stimulate osteogenesis and promote the integration of bone tissue through inorganic transformation [18], biosilica derived from marine sponges would have therapeutic potential to mineralize bone inorganic matrix. Although several studies have confirmed that spicules mixed with commercial inorganic bone substitutes could promote bone regeneration in vitro and in vivo [19,20], there were few studies on how spicules induce osteogenic differentiation of stem cells and demonstrated effects in vivo through a specific mechanism [21]. Therefore, its osteoinductive mechanism with clear assessments should be explored.

**Fig. 1.**
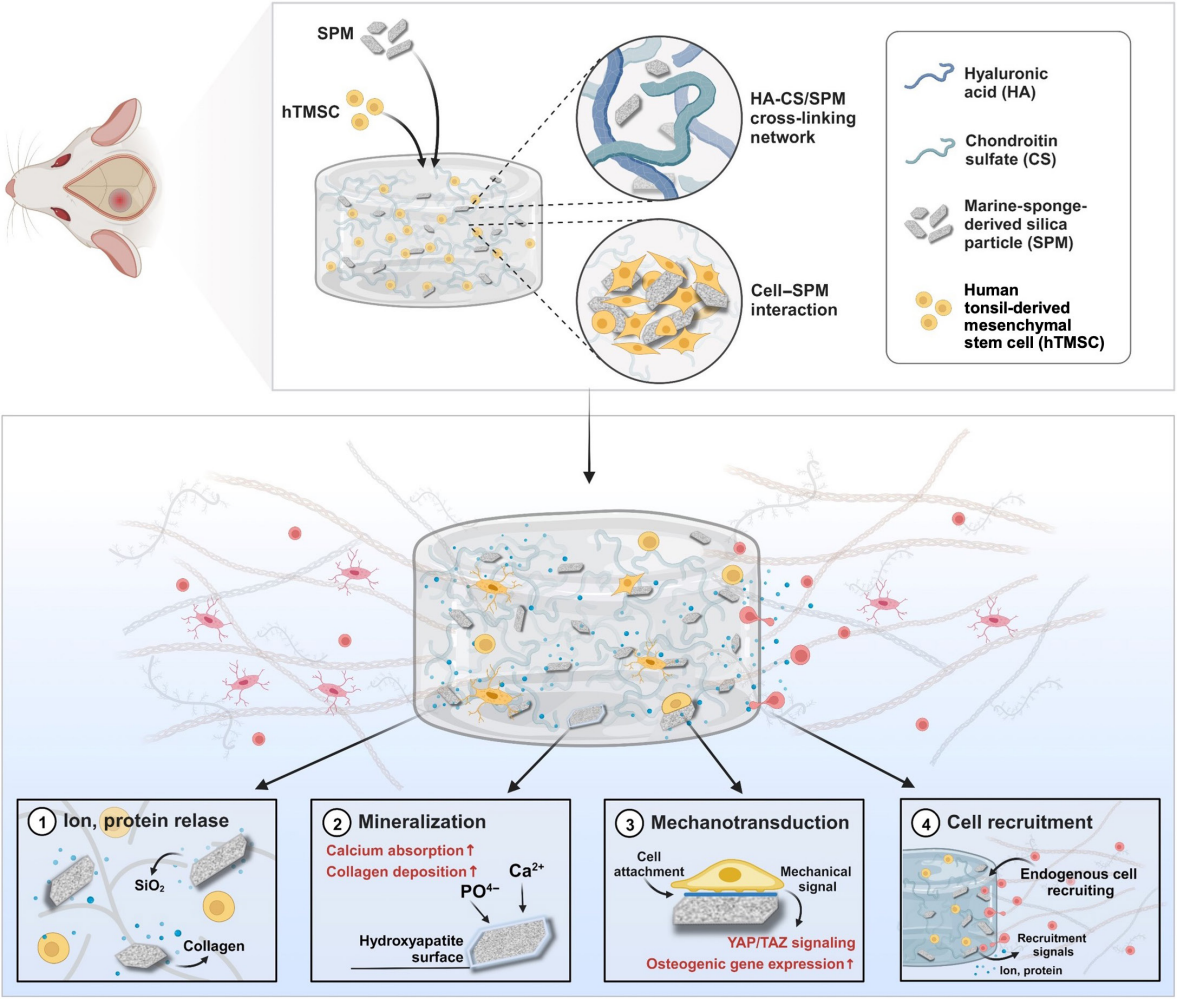
Schematic illustration of the mechanism of vascularized new bone formation by SPM.

Here, the objective of this study is to propose the osteogenic mechanism of spicule microparticles (SPMs) and investigate new bone formation in the bone defect model (Fig. 1). Scanning electron microscopy (SEM) revealed smooth, needle-like spicules with uniform size and silica composition, as confirmed by energy-dispersive x-ray spectroscopy (EDS) and x-ray powder diffraction (XRD). The osteogenic properties of spicules were explored by grinding them into SPM. SPM exhibited mineralization in simulated body fluid (SBF) over 7, 14, and 21 d, with SEM showing mineral deposition and HAP crystal formation. In vitro, osteogenic responses of SPM were validated by coculturing human tonsil-derived mesenchymal stem cells (hTMSCs) with SPM. Real-time polymerase chain reaction (PCR) analysis showed up-regulated osteogenic genes, demonstrating heightened osteogenic differentiation facilitated by SPM. Adhesion of hTMSCs and activation of the Yes-associated protein 1 (YAP) signaling pathway were observed in the presence of SPM. Cell morphology revealed alignment along SPM, indicating a 3-dimension-like environment. YAP/transcriptional coactivator with PDZ-binding motif (TAZ) signaling analysis demonstrated increased genetic expression, confirming SPM’s effective induction of osteogenesis through a mechanochemical microenvironment. In vivo, the bone regeneration potential of hydrogel-incorporating SPM (HC_SPM) was assessed in a mouse cranial defect model. Micro-computed-tomography (micro-CT) imaging showed enhanced bone formation in the HC_SPM group, with significant improvements in bone parameters. Histological analysis confirmed successful bone regeneration, supporting the promising in vitro results.

## Materials and Methods

### Preparation and characterization of SPM

In this study, we used demosponge-derived spicules (UNIZ LAB, Incheon, South Korea) originating from Ukraine. To make SPM, we ground spicules in liquid nitrogen environments. Briefly, the spicule was frozen in liquid nitrogen for 1 h. After 1 h, the frozen spicule was ground into microsized particles using a mortar and pestle. To reduce the unevenness of spicules, we used grinding, followed by sieving through the mesh. Then, the ground spicule was sieved step by step through a sieve of 25- and 10-μm pore size to remove foreign substances and prepare a uniform spicule. The final product (SPM) was sterilized by ultraviolet treatment for 1 h before in vitro osteogenesis. To observe the structure of the SPM, the SPM was fixed with carbon tape and coated with platinum using a sputter coater. The surface and pores of SPM were observed using a field-emission SEM (JSM-6700F, JEOL). The elemental composition of the SPM was then analyzed by EDS. The crystalline phase and internal structure of the SPM were recorded using XRD (miniflex 600 x-ray, rangaku) (40 kV, 15 mA, Cu Kα radiation, λ = 1.5418 Å). The surface chemical properties of SPM were analyzed by x-ray photoelectron spectroscopy (XPS) (K-Alpha+XPS system, Thermo Fisher Scientific). Photoelectron spectra were recorded using an Al Kα x-ray source (*hv* = 1,486.6 eV, 12 kV, 72 W, 400-μm spot size). The collagen content and release capacity of SPM were confirmed through the release characteristics of collagen over time. The released collagen was analyzed for its soluble form using the Sircol soluble collagen assay (Biocolor, Carrickfergus, UK) according to the manufacturer’s instructions. Briefly, 10 mg of SPM was incubated in 10 ml of 0.5 M acetic acid containing 1 wt% (w/v) of pepsin for 2, 4, 8, 12, 24, and 48 h at 4 °C with agitation. The supernatant (pepsin-soluble collagen) was collected through centrifugation at each time point. Similarly, soluble collagen was also cultured in phosphate-buffered saline (PBS) (pH 7.4). Afterward, 1 ml of collagen-binding dye was added to the collected supernatant and mixed for 30 min. After centrifugation, the pellet was suspended in 1 ml of alkaline reagent, and the absorbance was measured at 540 nm using a standard spectrophotometer.

### In vitro apatite formation assay

To measure apatite formation by SPM, the SPM in SBF solution was analyzed. Briefly, the SPM were immersed in an SBF solution containing 7.996 g of NaCl, 0.350 g of NaHCO_3_, 0.224 g of KCl, 0.228 g of K_2_HPO_4_·3H_2_O, 0.305 g of MgCl_2_·6H_2_O, 0.278 g of CaCl_2_, 0.071 g of Na_2_SO_4_, and 6.057 g of (CH_2_OH)_3_CNH_2_ in 1 l of deionized water. The SBF solution was adjusted to a final pH of 7.40 with 1 N of HCl. The SPM in SBF solution was incubated at 37 °C for 7, 14, and 21 d, washed with deionized water, and vacuum-dried. After drying, apatite formation on the SPM’s surface was observed using field-emission SEM. The elemental composition was analyzed by EDS mapping to confirm the phosphoric acid and calcium ion deposition. To confirm the phase change of the sample, XRD diffraction patterns were recorded to analyze the chemical composition and crystal structure. To analyze the surface chemical characteristics of the crystals formed in SPM, each ion was detected using XPS.

### In vitro osteogenic differentiation

We used hTMSCs to evaluate osteogenesis by SPM. hTMSCs were harvested from the tonsil tissue of the tonsillectomy patient. First, hTMSCs were seeded in 12 Transwells at a density of 1 × 10^5^ cells/ml, and after cell attachment, the SPM were cocultured with cells by insert (0.3 μm; SPLInsert Hanging, SPL). Osteogenic differentiation medium was prepared by adding 100 nM dexamethasone, 10 mM glycerol-2-phosphate, l-ascorbic acid (50 μg/ml), and 1% antibiotic–antimycotic to high-glucose Dulbecco’s modified Eagle’s medium containing 10% fetal bovine serum and 1% penicillin–streptomycin, and the differentiation medium was replaced every other day. Cells were subcultured when they reached the plate’s 70% to 80% confluency.

### Alkaline phosphatase and Alizarin Red S staining

To confirm osteogenic differentiation, all samples were fixed with 4% paraformaldehyde for 10 min before staining. To evaluate alkaline phosphatase (ALP) activity 7 d after induction of bone formation, the fixed cells were washed with PBS, stained with BCIP/NBT (5-bromo-4-chloro-3′-indoliphosphate *p*-toluidine salt/nitroblue tetrazolium chloride) for 30 min, and washed with PBS. ARS solution (2%) was prepared for Alizarin Red S (ARS) staining. First, after dissolving 2% (w/v) of ARS in deionized water, it was adjusted to a final pH of 4.1 to 4.3 using NH_4_OH. On day 14 of osteogenesis induction, the samples were fixed, washed with deionized water, stained with 2% ARS for 30 min, and washed with distilled water to remove excess ARS.

### Observation of cell adhesion

Cell adhesion to SPM was confirmed through fluorescently labeled SPM. Briefly, 10 mg of SPM was reacted with 0.2 ml of 15 mM 3-aminopropyltriethoxysilane, and unreacted 3-aminopropyltriethoxysilane was removed through ethanol washing. Afterward, 3 mM Rhodamine B isocyanate (RITC) was added to a final concentration of 3 mM, reacted for 10 min, and washed with distilled water to prepare RITC-labeled SPM. RITC-labeled SPM and hTMSCs were mixed in culture and observed through a fluorescence microscope 24 h later. Before staining, cells were fixed with 4% paraformaldehyde for 10 min, washed with PBS, and then permeabilized with 0.1% Triton X-100 in PBS for 10 min. After washing with PBS, the cells were blocked with 1% bovine serum albumin in PBS for 40 min. Subsequently, it was treated with 0.2 μM Phalloidin 488 and Hoechst 33342 (2 μg/ml) for 30 min in the dark. The group without SPM and the nonfluorescently labeled SPM group also proceeded in the same way.

### Assessment of mechanotransduction of hTMSCs

To investigate the mechanistic involvement of SPM in osteogenic differentiation, we performed mixed cultures of hTMSCs and SPM. After culturing with various concentrations of SPM for 7 and 14 d, it was confirmed through the abovementioned ALP and ARS activities. In addition, the ability of SPM to regulate mechanotransduction was investigated through the YAP/TAZ signaling pathway. hTMSCs were seeded on gelatin-coated tissue culture plate (TCP) and mixed cultured with SPM, and gelatin-coated TCP without SPM was used as a control. To investigate only the effect on the mechanical stiffness of the substrate, the same osteogenic differentiation induction medium was used.

### Real-time PCR analysis

Total RNA was isolated and relative mRNA expression levels were determined using quantitative reverse transcription PCR. Bone-related genes [runt-related transcription factor 2 (RunX2), ALP, collagen type 1 (COL1), and osteocalcin (OCN)] and mechanotransduction-related genes [YAP, TAZ, and connective tissue growth factor (CTGF)] were detected by amplifying cDNA with SYBR Green. All samples were normalized by the housekeeping gene GAPDH. Primer lists are shown in Table S1.

### Synthesis and characterization of tyrosinase from *Streptomyces avermitilis*, tyramine-conjugated hyaluronic acid, and tyramine-conjugated chondroitin sulfate

Recombinant tyrosinase (Ty) from *Streptomyces avermitilis* was synthesized and extracted following previous studies [22]. We used tyramine-conjugated hyaluronic acid (HA_T) and chondroitin sulfate (CS_T) as scaffolds for SPM. To synthesize HA_T and CS_T (HC), we used 1-ethyl-3-(3-dimethylaminopropyl)carbodiimide (EDC)/*N*-hydroxysuccinimide (NHS) coupling chemistry. Briefly, sodium hyaluronate was dissolved in distilled water to prepare a 1% (w/v) of hyaluronic acid solution. While stirring, *N*-hydroxysulfosuccinimid (TCI, Tokyo, Japan) and EDC·HCl (Thermo Fisher Scientific, MA, USA) were added to the hyaluronic acid solution in a 1:1 molar ratio with COOH of hyaluronic acid. Afterward, tyramine hydrochloride (Alfa Aesar, MA, USA) was added in a 1:1 molar ratio to hyaluronic acid. After the overnight reaction, the product was dialyzed against deionized water for 3 d. The synthesized HA_T was lyophilized and kept at −20 °C until further the experiment. Synthesis of CS_T proceeded in the same way.

^1^H spectrum was recorded for HC using a nuclear magnetic resonance (NMR) (400 MHz; MR400 DD2, Agilent) spectrometer to confirm the conjugation of tyramine on the hyaluronic acid and chondroitin sulfate. HC were dissolved in deuterium oxide (100 atom % of D; Acros Organics), respectively, and added to a 5-mm NMR tube. Analysis was performed at 23 °C, 400-MHz frequency, and 9.4-T magnetic field strength conditions. Spectra were processed with Agilent VnmrJ software.

The synthesized HA_T was dissolved in PBS at 2% (w/v). In addition, CS_T was dissolved in PBS at 1%, 2%, and 4% (w/v). Then, the HC solutions were sterilized with a 0.22-μm syringe filter and mixed in a 1:1 ratio to prepare a hydrogel precursor. The precursor solution was mixed, and Ty (20 uM) was added to the precursor solution to induce enzymatic cross-linking. After 15 min, HA_T with CS_T hydrogel (HC hydrogel) was fully cross-linked. The HC-hydrogel-containing SPM (HC_SPM) was also performed in the same manner as described above and was prepared by adding SPM to the hydrogel precursor solution.

To measure the swelling ratio (*Q*), the HC hydrogel was lyophilized, and the dry weight (*W*_d_) was measured. After soaking the dried hydrogels (*n* = 3) in PBS for 24 h, the wet weight (*W*_s_) was measured after swiping residual PBS with Kimwipes. The swelling ratio was calculated using Eq. 1.

$$Q=\left(W_{s}-W_{d}\right)/\left(W_{d}\right)$$ (1)

To confirm the biomineralization of the HC hydrogel, each sample was immersed in SBF, washed with deionized water, and lyophilized. The lyophilized samples were then mounted on SEM mounts with carbon tape. Samples were coated with platinum in a vacuum. Images were observed through an SEM.

### Cell viability assay

To analyze cytotoxicity, mouse myoblasts (C2C12) were seeded at a density of 1 × 10^5^ cells on an HC hydrogel (diameter, 8 mm; height, 2 mm). After attachment, high-glucose Dulbecco’s modified Eagle’s medium containing 10% fetal bovine serum and 1% penicillin–streptomycin was added to the cells on the hydrogel. After 12 and 24 h of incubation, cytotoxicity was measured using a Live/Dead assay kit (Invitrogen) following the manufacturer’s instructions. The cells on HC hydrogel were stained for 15 min in the dark and washed with PBS. The SPM group was also measured in the same way. Images were observed through a fluorescence microscope (LS40Plus, LEAM Solution). Cell proliferation rate was evaluated by Alamar Blue assay. Briefly, hTMSCs were seeded at a density of 1 × 10^4^ in a 96-well plate, and SPM was added at concentrations of 0 and 4 mg/ml for each group. At 0, 1, 2, 5, 7, and 10 d after culture, Alamar Blue reagent (Invitrogen) was added at 10% concentration to the medium for each time point and incubated for 1 h at 37 °C and 5% CO_2_. Absorbance was then recorded at 570 nm using a plate reader. All groups were calibrated with control samples with staining solution added to empty wells (wells without cells).

### In vivo animal test

Animal surgery was conducted following the guidelines for the Care and Use of Laboratory Animals by the Seoul National University (SNU-230718-8). Eight-week Balb/c mice were prepared for surgery. After the mouse was anesthetized, the hair on the skull was shaved, and the skin was incised. Then, a defect with a diameter of 4 mm was drilled on the cranial, and the prepared hydrogel was implanted. Groups consist of negative control, SPM, only hydrogel, and SPM containing hydrogel. SPM was dispersed in PBS at 4 mg/ml and applied to the defect area. The skin was sutured, and the progress was observed for 8 weeks. After 8 weeks, mice were sacrificed using CO_2_, and cranial samples were harvested.

### Micro-CT analysis

All specimens were harvested and promptly immersed in 4% paraformaldehyde for subsequent histopathological, as well as micro-CT assessments. The cranial underwent scanning using the micro-CT scanner (Quantum GX2, PerkinElmer). Subsequently, a precise threshold was applied to segment the reconstructed dataset. The resultant images were reconstructed into 3-dimensional (3D) representations utilizing the ReCon Micro-CT software and 3D Slicer.

### Histological analysis

The specimens were fixed in 4% paraformaldehyde and decalcified in 20% EDTA (Bio-Rad) at pH 7.4 for 7 d. The segments were dehydrated and embedded in the paraffin block (Leica Biosystems). Tissues were sectioned with a 10-μm thickness, deparaffinated with xylene, gradually hydrated with ethanol, and stained with hematoxylin and eosin (H&E) and Masson’s trichrome staining (MTC) (Abcam) with the manufacturer’s guidance for light microscopic analysis. Images were captured using the microscope (Nikon Eclipse Ti2).

### Statistical analysis

All quantitative data were expressed as means ± SD, and each experiment was performed using 3 samples. Statistical significance was determined using Student’s *t* test and 2-way analysis of variance (ANOVA). A *P* < 0.05 was considered statistically significant (*P < 0.05, **P < 0.01, and ***P < 0.001).

## Results

### Characterization of spicules from marine sponge

**Fig. 2.**
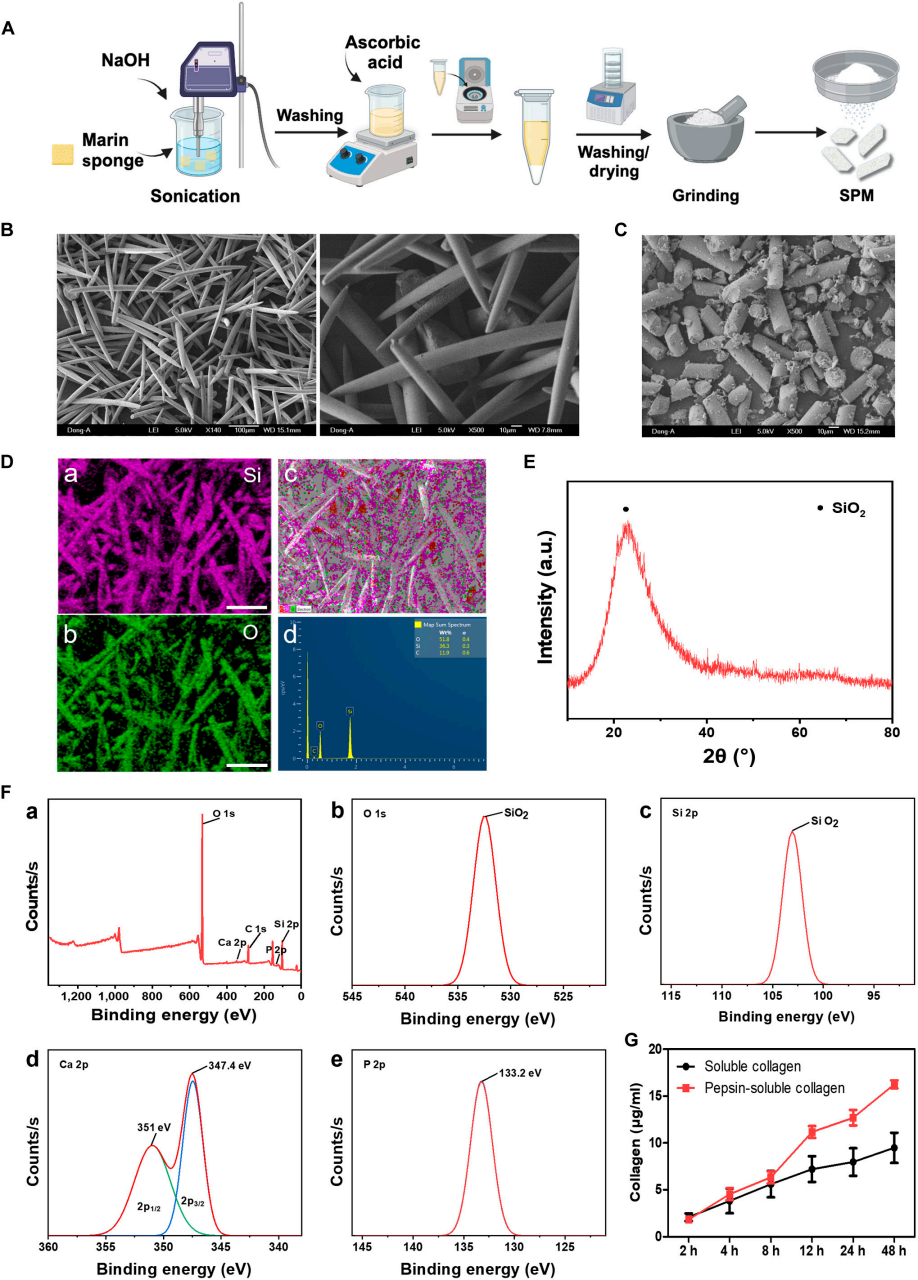
Characteristics of marine-sponge-derived spicules. (A) Schematics for spicule extraction and SPM fabrication. (B) SEM image of the surface and needle shape of spicules. Scale bars, 100 μm (left) and 10 μm (right). (C) SEM image of the SPM. Scale bar, 10 μm. (D) EDS analysis of SPM. (a and b) EDS maps of Si and O. Scale bars, 100 μm. (c) Overlap of the EDS mapping of the Si and O distribution. (d) EDS signal spectra of the (B). (E) XRD analysis of the SPM SiO_2_ crystal phase structure. (F) XPS analysis of surface. (a) XPS survey spectrum of SPM surface. (b and c) O 1s and Si 2p spectra showing O and Si bounding states. (d) Ca 2p spectrum. (e) P 2p spectrum. (G) Soluble collagen contents in SPM; soluble collagen (soluble collagen and pepsin-soluble collagen) released over time. Data are presented as means ± SD (*n* = 3). a.u., arbitrary units.

In this experiment, spicules were extracted from marine sponges, especially Demospongiae. As depicted in Fig. 2A and Fig. S1, a lyophilized sponge was treated with protease (100 U/ml) from the bovine pancreas to remove organic matter. Then, the dissolved sponge solution was centrifuged to obtain pure spicules. The size and morphology of the fabricated spicules were analyzed using SEM. The spicules exhibited a smooth surface and uniform size needle-like shape with an average length of approximately 200 to 250 μm and a width of 10 μm (Fig. 2B). The ground spicules called SPMs have also similar morphology compared to spicules (Fig. 2C). EDS was used to further analyze the elemental composition of spicules (Fig. 2D). EDS mapping confirmed that spicules consisted of silica oxide (SiO_2_). XRD results indicated a diffraction peak at around 20° to 30° θ, characteristic of amorphous silica, which was consistent with the EDS mapping results (Fig. 2E). We also evaluated elements of spicules using XPS survey spectra (Fig. 2F). For O 1s and Si 2p high-resolution spectra, the single chemical structure was observed at a binding energy of 532.8 and 103.5 eV, respectively. In addition, trace amounts of calcium and phosphate ions were detected at 347.4, 351, and 133.2 eV, respectively (Fig. 2F). This XPS data indicated that spicules consist of SiO_2_ mixed with trace amounts of calcium and phosphate ions. Next, to confirm the presence of collagen in SPM, we evaluated its release profile under PBS (pH 7.4) and pepsin treatment (Fig. 2G). Collagens in SPM were confirmed to be released under both conditions, revealing their solubility properties. These observations not only demonstrate the presence of collagen in SPM but also indicate that the soluble form of collagen can achieve sustained release due to its solubility properties under physiological conditions. This integrated composition holds the potential to create an environment conducive to bone regeneration, owing to the continuous release of collagen and the synergistic effects of essential minerals, including silicon, calcium, and phosphorus.

### HAP formation on spicules

**Fig. 3.**
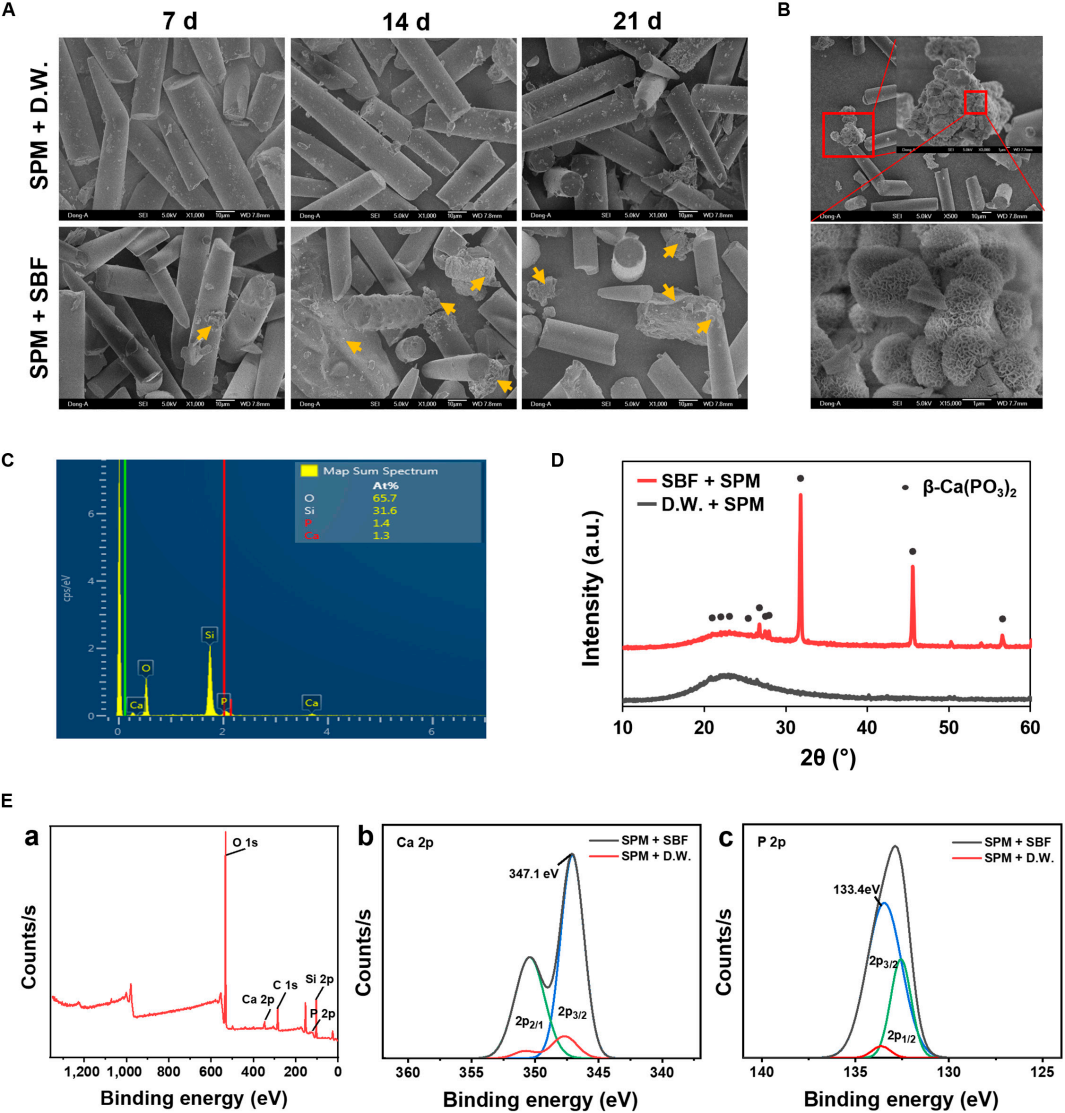
Evaluation of SPM biomineralization ability. (A) SEM image of SPM after immersing in SBF solution for 7, 14, and 21 d. Yellow arrows indicate mineralized areas. Scale bars, 10 μm. (B) SEM image of HAP formed on the SPM surface. (C) Characterization of the chemical composition of SPM immersed in SBF through EDS analysis or EDS signal spectra of the (B) EDS spectrum shows the generated Ca and P ions. (D) XRD analysis of SPM immersed in SBF and SPM immersed in distilled water (D.W.). (E) XPS analysis of surface. (a) XPS survey spectrum of the SPM surface immersed in SBF. (b and c) Ca 2p and P 2p spectra showing Ca and P bounding states of generated HAP.

Next, we evaluated the osteogenic properties of spicules. As the increase in surface area of inorganic particles can enhance osteogenic mineralization in vivo, we ground spicules to obtain SPMs. This process not only reduces the particle size (Fig. S2) but also increases the surface area, facilitating greater cell attachment sites and enhanced ion deposition. Furthermore, the increased surface area resulting from grinding provides more opportunities for calcium and phosphate adsorption, thereby promoting ion and protein release [23]. Although the size of SPM may be heterogeneous and the extent of osteogenesis may vary depending on the SPM size, it is considered suitable for the analysis of the osteogenic mechanism of stem cells, the primary aim of the current study. To simulate the biomineralization process of SPM within the body, we confirmed the formation of HAP in SBF [24,25]. The mineralization capacity of SPM was assessed by incubating SPM in SBF at 37 °C for 7, 14, and 21 d, followed by SEM observation of the particle surfaces at each time point (Fig. 3A). The mineral deposition was observed on the SPM surfaces in the SBF-treated groups at all time points (7, 14, and 21 d), in contrast to the distilled-water-treated group. SEM images also revealed the formation of cauliflower-like HAP crystals on the SPM surfaces (Fig. 3B). SBF solution mimics the human body fluid by containing calcium, phosphate, and other ions. SPM interacts with these ions to form HAP nuclei. Surrounding calcium and phosphate ions then deposit onto these nuclei, resulting in the formation of HAP crystals on the surface of SPM. EDS mapping and XRD analysis showed the emergence of new β-Ca(PO_3_)_2_ diffraction peaks in the SBF-treated group, indicating the formation of calcium phosphate on the SPM surface (Fig. 3C and D). From the XPS spectra survey, the binding energies corresponding to Ca 2p_3/2_ and P 2p_3/2_ were 347.1 and 133.4 eV, respectively (Fig. 3E). Ca 2p_3/2_ indicated calcium bonds related to HAP, and P 2p_3/2_ attributed to P–O bonds of HAP. These results demonstrated that SPM can be transformed into an HAP-like structure, called in vivo biomineralization.

### In vitro osteogenic response of SPMs

**Fig. 4.**
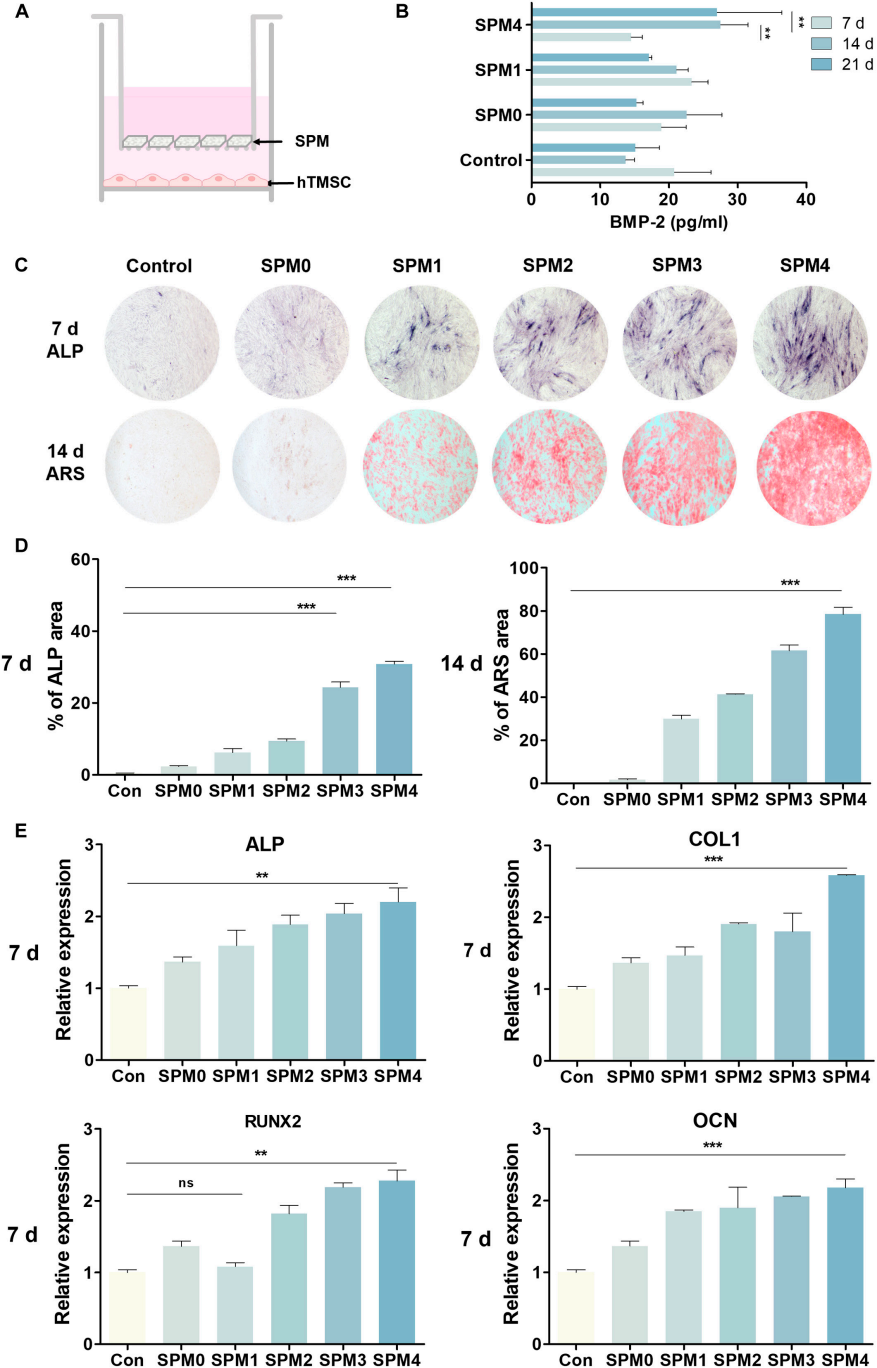
In vitro osteogenic assessment for SPM. (A) Schematic of SPM and cell coculture. (B) BMP-2 ELISA analysis of hTMSCs cocultured with SPM for 7, 14, and 21 d. (C) ALP and ARS staining images of hTMSCs cocultured with SPM for 7 and 14 d. (D) Quantification of ALP and ARS deposition areas. (E) Relative osteogenic gene expression in hTMSCs cocultured for 7 d. Data are presented as means ± SD (*n* = 6). ns > 0.05, **P < 0.01, and ***P < 0.001.

We evaluated the cell viability and proliferation rate of hTMSCs affected by SPM (Fig. S3). hTMSCs possess various differentiation capabilities similar to mesenchymal stem cells derived from other tissues following a common lineage for mesodermal differentiation [26]. Furthermore, hTMSCs have been studied for their outstanding osteogenic potential validated in several studies and tested in bone disease models such as osteoporosis, osteomyelitis, and cranial defects. When hTMSCs were treated with SPM (4 mg/ml), no cell toxicity was observed for up to 24 h (Fig. S3A and B). In both the control group without SPM and the experimental groups with varying concentrations of SPM (1, 2, 3, and 4 mg/ml), cell proliferation was observed (Fig. S3C); however, the proliferation rate was relatively lower in the SPM groups compared to the control group. These results suggest that differentiation induced by SPM may influence cell proliferation, as evidenced by the lower proliferation rate in the SPM groups compared to the SPM-absent control group. The results indicate a tendency for stem cells to differentiate rather than proliferate under the influence of SPM and osteogenic medium [27]. The osteoinduction of SPM was validated by coculturing hTMSCs with SPM using a Transwell (Fig. 4A). hTMSCs cultured in a growth medium were used as the negative control (control group), while hTMSCs grown in an osteogenic medium without SPM served as the positive control (SPM 0 mg/ml group). As bone differentiation progresses, the expression of bone-specific protein BMP-2 (bone morphogenetic protein-2), which is known to induce osteoblast differentiation in cell types of mesenchymal stem cells, increases in stem cells [28]. To investigate the expression of BMP-2, the hTMSCs were cocultured with, SPM for 21 d, and the BMP-2 expression level in the cell culture medium on the 7th, 14th, and 21st days was verified by BMP-2 enzyme-linked immunosorbent assay (ELISA) (Fig. 4B). In the high-concentration SPM group, the BMP-2 secretion was retained over 21 d, indicating that SPM would sustain consistent induction of osteogenic differentiation of stem cells.

Next, the osteogenic response of SPM to hTMSCs was visualized (Fig. 4C and D). First, ALP staining was performed on the 7th day of coculture to evaluate the ALP activity expressed in the early stage of osteogenic differentiation, and ARS staining was performed on the 14 d to confirm calcium deposition. ALP levels tended to increase with increasing SPM contents, compared to the control group, and the highest activity was shown in the SPM4 group. In the case of ARS staining, it was confirmed that the calcium deposition increased in a concentration-dependent manner as increasing SPM content.

Then, we quantified osteogenic gene expression ALP, OCN, COL1, and RUNX2 via real-time PCR analysis (Fig. 4E). Following 7 d of coculture with SPM, all SPM-treated groups exhibited up-regulated osteogenic genes compared to the control group. Significantly increased gene expression was observed in the SPM-treated groups relative to the negative control, with the increase being statistically significant for most genes compared to the positive control. As the concentration of SPM increased, gene expression was up-regulated, with the highest expression observed in SPM4. We also confirmed the expression of RUNX2 using immunostaining on the 7th day (Fig. S4). These results elucidate that SPM facilitates osteogenic differentiation in hTMSCs by releasing Si ions crucial for collagen synthesis along with Ca and P ions essential for mineralization. Consequently, SPM can promote stem cell osteogenic differentiation by creating a favorable microenvironment for bone mineralization and collagen synthesis.

### Adhesion of hTMSCs on SPM and activation of YAP/TAZ signaling pathway

**Fig. 5.**
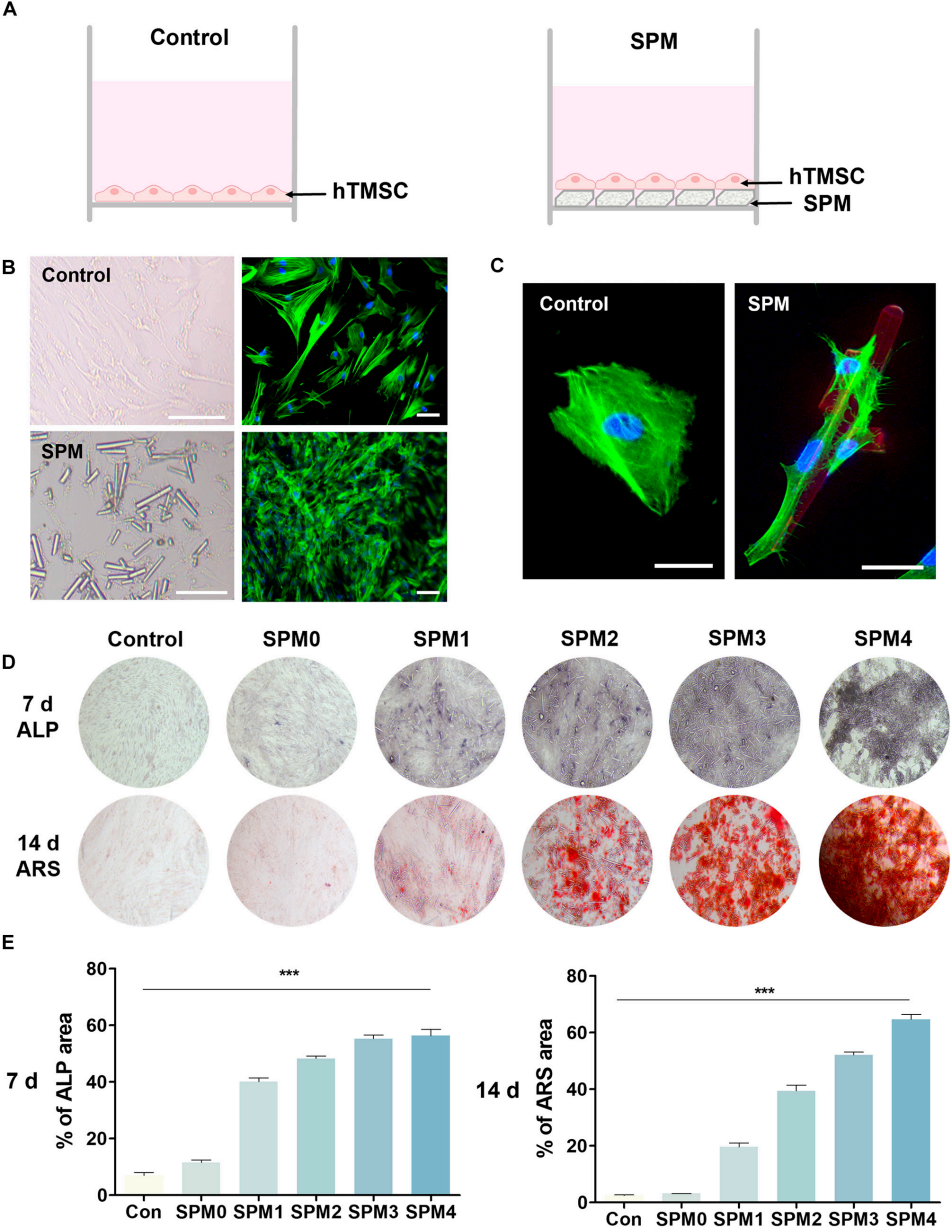
Effect of SPM on adhesion and differentiation of hTMSCs. (A) Schematic of the culture of hTMSCs. Control, only hTMSCs. (B) Image of adhesion pattern of hTMSCs with and without SPM. Control group, without SPM. Scale bars, 50 μm. (C) Morphological image of hTMSCs cultured with RITC-labeled SPM. Red, SPM; green, F-actin; blue, nucleus. (D) ALP and ARS staining images of hTMSCs mixed cultured with SPM for 7 and 14 d. (E) Quantification of ALP and ARS deposition areas. Data are presented as means ± SD (*n* = 7). ***P < 0.001.

To investigate the role of spicules in mechanical signal transmission, we observed the adhesion and spreading patterns of hTMSCs (Fig. 5A). As shown in Fig. 5B, the cell attachment pattern of hTMSCs on SPM exhibited a morphology that grew along the surface of the SPM, showing a distinct difference compared to groups without SPM. Observing cell morphology, we noticed that cells aligned themselves along the SPM, mimicking a 3D environment. The morphology of cultured hTMSCs was further assessed by staining the cytoskeleton and nuclei, which was observed with, RITC-labeled SPM (Fig. 5C). The cytoskeleton of hTMSCs showed the alignment of actin filaments in the direction of SPM growth. When the number of integrin protrusions on the surface of hTMSCs was quantified on the SPM surface, it increased more than fourfold compared to hTMSCs attached to TCP (Fig. S5). This indicates that hTMSCs adhere to the SPM due to increased integrin expression.

Next, a mixed culture of hTMSCs and SPM was performed to confirm the effect of direct contact with SPM. In the results of ALP and ARS staining, an increase in staining intensity and area was observed on the basis of the concentration of SPM. The most distinct staining was observed in the SPM4 group, indicating higher ALP and ARS expression compared to the results of the conventional Transwell coculture. In addition, when performing mixed culture with SPM, there was a significantly greater tendency for hTMSCs to adhere and grow on the SPM surface compared to the TCP substrate. As a result, darker staining of ARS and ALP was observed around the SPM, rather than on the TCP substrate (Fig. 5D and E). On the other hand, at a low concentration of SPM (SPM1 group), the expressions of both ARS and ALP were relatively lower than that of high concentration groups, which might be attributed to lower cell attachment efficacy on SPM. It demonstrated the high cellular binding between SPM and hTMSCs, as observed in Fig. 5B and C via F-actin staining.

**Fig. 6.**
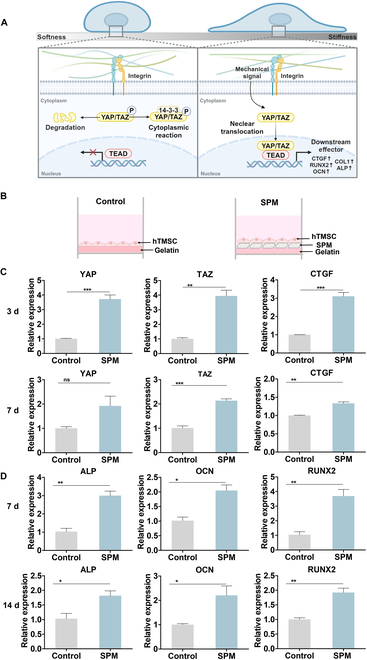
Effect of SPM on activation of YAP/TAZ signal pathway. (A) Related YAP/TAZ signal pathway mechanism in the mechanotransduction. (B) Schematic of the culture of hTMSCs. Control, gelatin-coated hTMSCs without SPM. (C) Real-time PCR analysis of YAP, TAZ, and CTGF genes in hTMSCs cultured for 3 and 7 d without and with SPM. (D) Relative osteogenic gene expression in hTMSCs cultured without and with SPM for 7 and 14 d. Data are presented as means ± SD (*n* = 3). ns > 0.05, *P < 0.05, **P < 0.01, and ***P < 0.001.

The Hippo signaling pathway is regulated by substrate stiffness. When the Hippo signaling pathway is inactive, YAP/TAZ is not phosphorylated, localizes to the nucleus, and associates with the transcription factor TEA domain transcription factor (TEAD) [29,30]. Thus, we investigated the mechanical environment provided by SPM based on the high binding between the preceding SPM and hTMSCs. We confirmed YAP/TAZ signaling, a mechanosensitive transcriptional regulator via SPM–cell binding. It then activates the SMAD1 pathway, which is important for osteogenic differentiation and regulates genes required for migration, proliferation, and differentiation (Fig. 6A) [31]. To investigate the influence of SPM’s mechanical rigidity on mechanotransduction, we compared cellular behavior in softness/stiffness environments using gelatin-coated TCP as a control group and gelatin-coated TCP with SPM conditions as the experimental group (Fig. 6B). We compared cell behavior in environments of gelatin-coated TCP alone and gelatin-coated TCP with SPM conditions, representing softness and stiffness, respectively. On the 3rd and 7th days, when examining the gene expression related to mechanotransduction, including YAP, TAZ, and CTGF, higher expression levels were observed on the 3rd day (Fig. 6C). Similarly, the expression of osteogenic genes (ALP, OCN, and RUNX2), downstream effectors of YAP/TAZ, also increased more on the 7th day compared to the 14th day (Fig. 6D). This suggests that stem cells undergo spreading, influencing the early stages of osteogenic differentiation on the 7th day by adhering to the SPM surface through F-actin and sensing mechanical cues. Subsequently, it demonstrated that the synergistic effect of the apatite-forming ability of SPM and its mechanical stiffness affected osteogenic differentiation. When comparing coculture and mixed culture of SPM with stem cells, mixed culture showed higher expression of ARS, ALP, and osteogenic genes (Fig. S6). This suggests that while SPM induces osteogenesis in both cases, mechanotransduction through direct interaction has a greater impact.

### Synthesis and characterization of HC_SPM

To administer SPM in vivo, we engineered a hydrogel composed of a blend of hyaluronic acid and chondroitin sulfate, cross-linked by Ty (Fig. S7A). The combination of hyaluronic acid and chondroitin sulfate polymers can synergistically enhance cell migration and attachment at the defect site and simultaneously adsorb calcium and phosphate ions that contribute to the formation of a bone mineral environment, facilitating bone formation. Under physiological conditions, Ty oxidizes tyramine to form quinone groups, which, due to their high reactivity, can enhance in vivo adhesion through shared bonding with abundant phenolic groups, amines, thiols, and imidazole groups in natural tissues. Ty’s enzyme–substrate specificity induces selective reactions, preventing unwanted side reactions and eliminating the need for harsh organic solvents or radical species required for chemical cross-linking. Hence, it is anticipated that Ty-mediated enzymatic hydrogels could be stably used under physiological conditions, enhancing biocompatibility with tissues such as cells and proteins [32]. Subsequently, HC were combined to form the HC hydrogel through a Ty-mediated oxidation reaction. ^1^H NMR spectra of HC revealed hydroxy group peaks between 6.8 and 7.5 parts per million (Fig. S7B), confirming successful tyramine conjugation. We identified the concentration that yielded the most suitable results in cross-linking by Ty following gross images of hydrogel, swelling ratio, and SEM images (Fig. S7C to F). Typically, hydrogel cross-linking by Ty results in a color change of the hydrogel from yellow to brown as the degree of cross-linking increases. Through this color change, the degree of cross-linking of the hydrogel can be indirectly evaluated. Although it is speculated that higher concentrations may yield even better outcomes, we chose to use the identified concentration to emphasize the osteogenesis effect mediated by SPM rather than the osteogenesis effect induced by the hydrogel as a carrier for SPM delivery. We selected the 1% (w/v) of HA_T mixed with 2% (w/v) of CS_T hydrogel for further experiments.

**Fig. 7.**
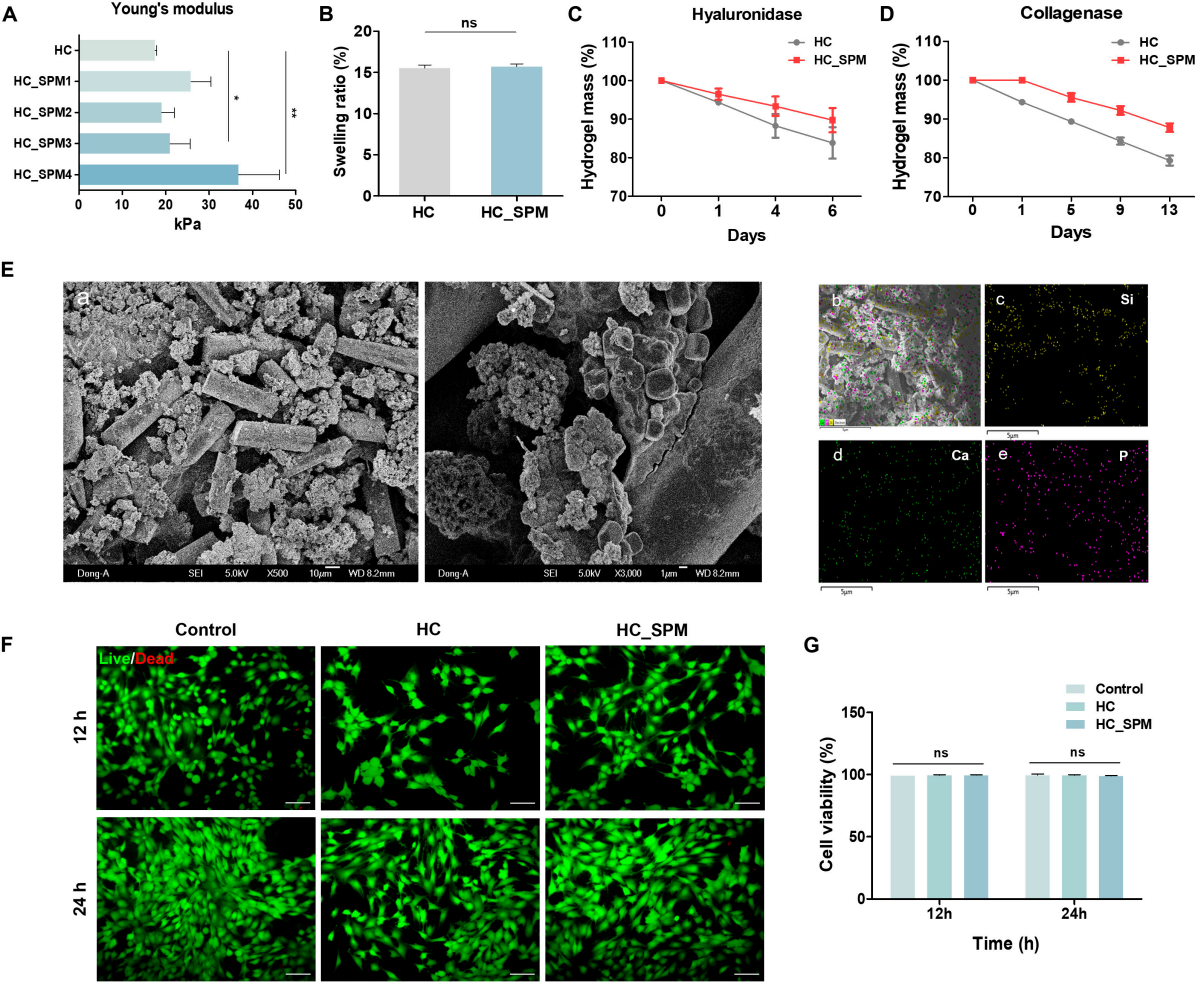
HC_SPM hydrogel characterization. (A) Young’s modulus of HC_SPM. Various concentrations of SPM (0, 1, 2, 3, and 4 mg/ml) were analyzed. (B) Analysis of hydrogel swelling rate after incubation in PBS for 24 h. (C) Enzymatic degradation of HC_SPM using hyaluronidase (10 U/ml) for 1, 4, and 6 d. (D) Enzymatic degradation of HC_SPM using collagenase (30 U/ml) for 1, 5, 9, and 13 d. (E) Image of HAP formation in SBF-immersed HC_SPM. (a) SEM image of HC_SPM surface morphology. Scale bars, 10 μm (left) and 1 μm (right). (b) Overlap of the EDS mapping of the Si, Ca, and P distribution. (c to e) EDS maps of Si, Ca, and P. Scale bars, 5 μm. (D) Analysis of hydrogel swelling rate after incubation in PBS for 24 h. (F) Cell viability as measured by Live/Dead assay of C2C12 cell cultured in hydrogels. Live cells were stained with calcein acetoxymethyl (AM) (green), and dead cells were stained with ethidium homodimer-1 (red). Scale bar, 50 μm. (G) Quantification of dead cells compared to live cells. Data are presented as means ± SD (*n* = 3). ns > 0.05, *P < 0.05, and **P < 0.01.

HC_SPMs were prepared by incorporating various concentrations (1, 2, 3, and 4 mg/ml) of SPM into HC hydrogel precursor solutions. On the basis of our previous research, silica-based particles can adsorb biopolymers such as hyaluronic acid and gelatin onto their surface area [33]. Enzymatic cross-linking is facilitated because the polymeric chains absorbed on the surface of silica-based particles have more chance of being oxidized by Ty. To assess the mechanical properties with increasing SPM concentration, we measured Young’s modulus through the slope of the stress–strain curve (Fig. 7A). The control group without SPM represented approximately 17.5 kPa, while it significantly increased with higher SPM content up to about 37 kPa in the SPM4. The swelling ratio did not show significant changes based on the presence of SPM (Fig. 7B). Furthermore, we conducted hydrogel degradation experiments with HC as the control group and HC_SPM4 as the experimental group, using both hyaluronidase and collagenase (Fig. 7C and D). Hyaluronidase mimics the matrix metalloproteinases produced by cells in the body. Collagenase was included to assess its impact on hydrogel degradation as collagen, contained in spicule, breaks down. In the experiments with hyaluronidase treatment, HC_SPM4 exhibited a delayed degradation tendency. However, both groups showed only up to approximately 20% degradation over 6 d. In the collagenase treatment experiments, hydrogel degradation reached up to 15% over a maximum of 13 d, indicating that spicule affects hydrogel formation.

On the basis of these experiments, the HC_SPM4 group was chosen for application in in vivo bone defects. In addition, when HC_SPM4 was treated with SBF solution, the formation of HAP was observed (Fig. 7E). This is attributed to the sulfate groups of chondroitin sulfate being able to adsorb ions, leading to enhanced HAP formation. Furthermore, enzyme-treated hydrogels demonstrated high biocompatibility, and HC_SPM also showed no observed cytotoxicity (Fig. 7F and G).

### In vivo bone regeneration potential of HC_SPM

**Fig. 8.**
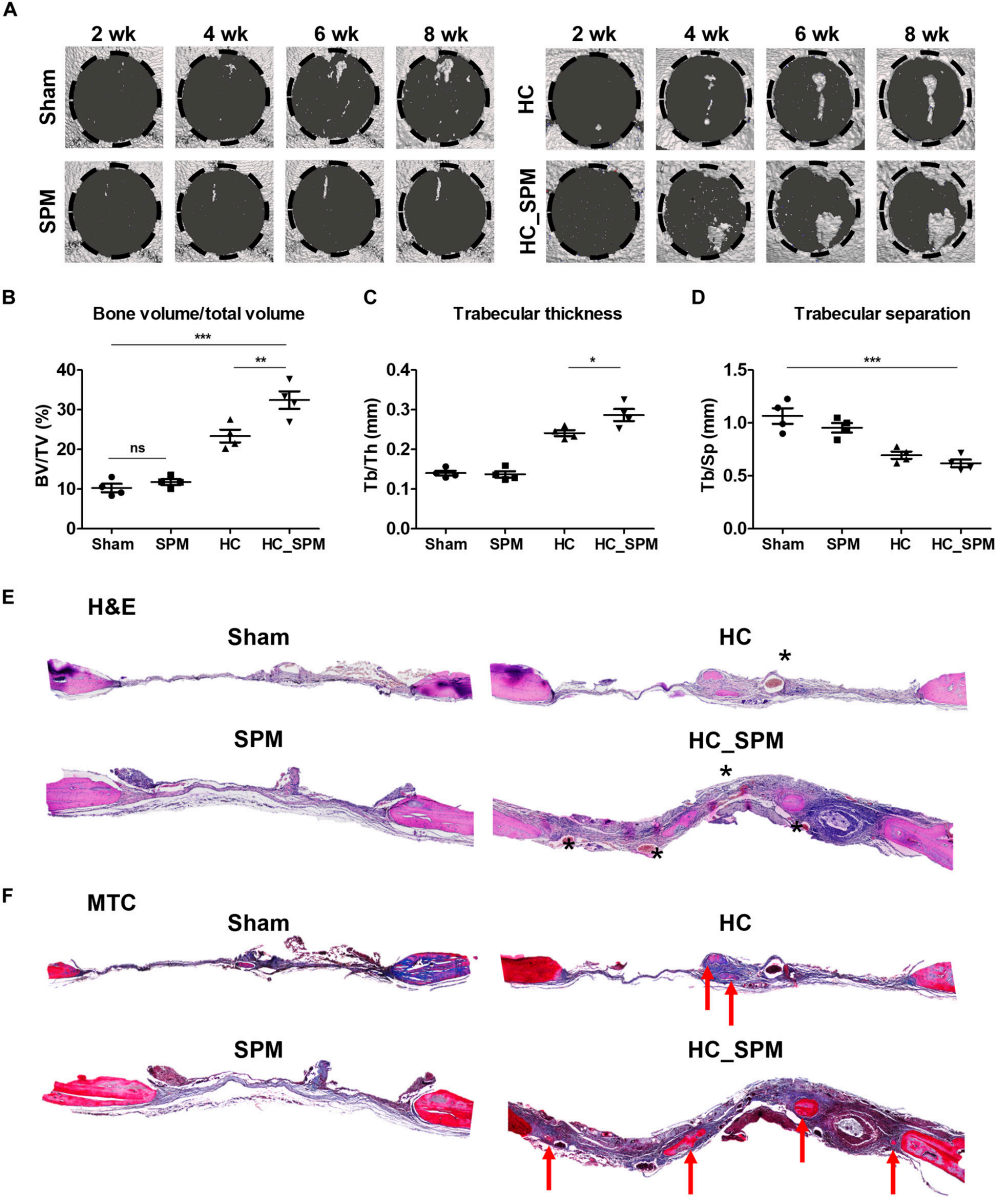
In vivo bone formation assessment of HC_SPM. (A) Micro-CT image of mouse calvaria defects at 2, 4, 6, and 8 weeks after implantation. The black circle indicates the surgical site. (B) BV/TV ratio of the calvaria tissue 8 weeks after implantation, as well as (C) Tb/Th and (D) Tb/Sp. (E) Histological analysis with H&E staining at 8 weeks after implantation. (F) Masson’s trichrome staining. Black asterisks indicate blood vessels, and the red arrows indicate new bone. Data are presented as means ± SD (*n* = 3). *P < 0.05, **P < 0.01, and ***P < 0.001.

A mouse cranial defect model was used to assess the bone regenerative effects of the HC_SPM. The experimental groups included a negative control, SPM, HC, and HC_SPM. The progression of bone regeneration was monitored using live micro-CT scans every 2 weeks until the mice were sacrificed after defect (Fig. 8A). In the CT images, the control and SPM groups exhibited minimal bone regeneration over the 8 weeks. Significant bone regeneration was not observed in the SPM group, and this is attributed to the rapid degradation of SPM in vivo, rendering the mechanism demonstrated in vitro ineffective. Conversely, increased bone formation was evident in the hydrogel and HC_SPM groups compared to the control group. Notably, the HC_SPM group demonstrated enhanced bone regeneration at the defect boundary, with the highest bone volume/total volume (BV/TV) ratio (HC versus HC_SPM, 23.37% versus 32.43%) (Fig. 8B). In addition, statistically significant differences were observed in bone parameters, particularly trabecular thickness (Tb/Th) and trabecular separation (Tb/Sp), indicating a significant osteogenic effect of the HC_SPM group (Fig. 8C and D). This is attributed to the encapsulation of SPM in the hydrogel, which can delay degradation by in vivo enzymes. Therefore, it is presumed that the biomineralization, ion release, and mechanotransduction observed in the in vitro results played a role in this context.

**Fig. 9.**
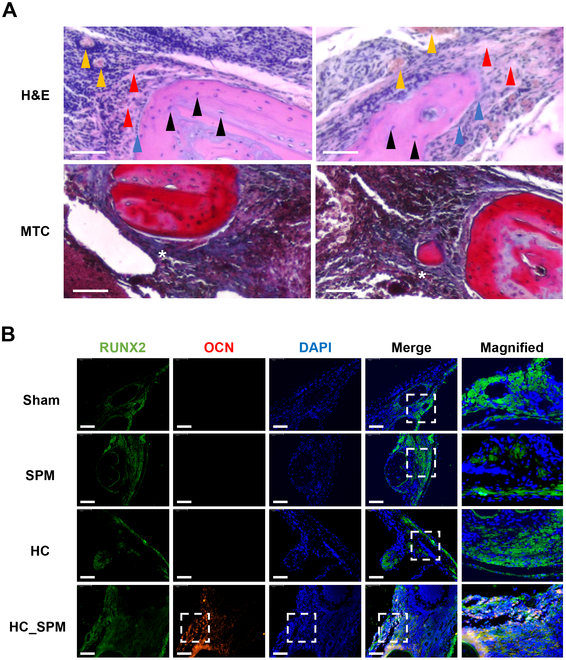
Bone formation effects of the HC_SPM in the calvarial defect model. (A) H&E staining magnified image of HC_SPM group in vivo calvarial defect model. Black, red, blue, and yellow arrowheads indicate osteocyte, osteoid, osteoblast, and vasculature, respectively; White asterisk indicates new bone. Scale bars, 20 μm. (B) Immunohistochemistry analysis of Runx2 and OCN expression in calvarial defect model. Scale bars, 100 μm. DAPI, 4′,6-diamidino-2-phenylindole.

Histological analysis of the cranial defect was conducted using H&E and MTC (Fig. 8E and F). Each image depicts the entire bone tissue of the defect, and a magnified view for each group was obtained from samples analyzed 8 weeks after surgery. The HC_SPM group exhibited densely composed collagen tissue (blue) with a thickness similar to the original bone. In particular, when comparing the hydrogel group to the HC_SPM group, the difference in collagen tissues between the groups was evident. Furthermore, in the HC_SPM group, a significant presence of red blood cells was observed. This could serve as evidence that SPM itself may induce neovascularization (Fig. 8E and F). The H&E image of the HC_SPM group distinguished morphological analysis of cells in the regenerated site and marked with arrows. A number of osteocytes and osteoblasts are observed in the regenerated bone of the HC_SPM group, and red blood cells indicate the presence of microvasculature around the regenerated bone (Fig. 9A). Immunohistochemical staining was performed using labeling for osteogenic factors (Fig. 9B). All groups showed Runx2 expression in the defect area, and there was no difference in the level of Runx2 between the sham, HC, and SPM groups. Interestingly, only the HC_SPM group shows OCN expression in many cells. This is consistent with our in vitro experiments showing that spicules assist osteogenic maturation and mineral formation during bone regeneration. Therefore, the SPM in the hydrogel promotes the focal adhesion of bone cells to the scaffold and induces mechanotransduction of osteoblasts, ultimately resulting in successful bone regeneration that aligns with the in vitro results.

## Discussion

The primary objective of this study was to investigate the osteogenic effects of marine-sponge-derived spicules, specifically pure silica dioxide mixed with proteins such as collagen, both in vitro and in vivo. Our hypotheses suggested 3 potential ways in which spicules could induce osteogenesis. First, spicules could undergo mineralization into an HAP form, termed osteoconduction, due to the presence of silica ions on their surface, which have the potential to enhance calcium absorption and promote collagen deposition. Second, silica ions released from spicules could initiate osteoinduction in native stem cells. Third, spicules could effectively serve as a site for cell adhesion, promoting osteogenesis through mechanotransduction.

To investigate these hypotheses, we used 2 main experimental approaches. First, SPM Transwell culture was utilized to examine indirect osteogenic effects through ions and proteins released from SPM. Second, the SPM coculture system was used to simultaneously assess direct cell contact and the resulting osteogenic effect. These methods were chosen on the basis of the specific role of the SPM being examined. Specifically, the Transwell setup allowed us to assess the osteoinductive effects mediated by ion and collagen release. In contrast, direct culture experiments aimed to elucidate the impact of endogenous cell migration and recruitment mediated by ion and collagen release, as well as the influence of spicule mechanical rigidity and its effects on the osteoconductive environment. This strategic approach aimed to comprehensively understand the osteogenic potential of spicules and their ability to modulate cell behavior in a tissue-like environment.

In the initial phase of our hypothesis testing, we observed that SPM induced biomineralization, forming an HAP-like structure under physiological conditions. Unlike industrial synthesis conditions (e.g., high temperature and extremes of pH and pressure), the in vivo biosynthesis of biosilica from marine sponge occurs within cells, involving silicon and the enzyme silicatein under physiological conditions [16,34]. This results in biosilica containing proteins such as collagen within its structure, possessing an amorphous architecture that leads to high biocompatibility and biodegradability. Data in Fig. 2 on the characterization of SPM incorporating collagen in a SiO_2_ matrix containing essential minerals such as calcium and phosphorus supplemented our hypothesis and highlighted the potential utility of SPM in promoting bone regeneration. Biosilica facilitates biomineralization at the inorganic–organic interface [35], allowing smooth ion adsorption, leading to the formation of HAP-like structures in SBF solution, starting from the 7th day.

Subsequently, the osteogenic differentiation of hTMSCs was evaluated with silicon ions and collagens released from SPM. Silicon ions induced osteogenic differentiation by activating the Wnt and extracellular-signal-regulated kinase (ERK) signaling pathways in endogenous stem cells. This activation led to the translocation of ERK and β-catenin into the cell nucleus, promoting the expression of osteogenic genes (ALP, OCN, RUNX2, etc.), ultimately facilitating osteogenic differentiation [36,37]. When coculturing SPM and hTMSCs using a Transwell system, an increase in the expression of COL1, ALP, OCN, and RUNX2 was observed because of the release of silicon ions from SPM. This increase was dose-dependent on the concentration of SPM, and the elevated expression of osteogenic genes was validated to affect BMP-2 protein expression in hTMSCs through ELISA. In addition, an increase in ALP and biomineralization was observed in ALP and ARS staining, respectively, depending on the concentration of SPM. This was attributed to the influence of silicon ions and collagen released from SPM. In addition, during the spicule grinding process, some SPMs were mixed in, and it is believed that these microparticles may directly bind to hTMSCs, influencing osteogenesis.

It was further demonstrated that when stem cells interacted with biosilica, the expression of α_V_β_3_ integrin increased, leading to the binding of stem cells to the biosilica surface [38,39]. The increased expression of α_V_β_3_ integrin activated the mitogen-activated protein kinase (MAPK) pathway, resulting in the nuclear translocation of ERK, c-Jun N-terminal kinase (JNK), p38, and osteogenic genes [40,41]. In mixed cultures of SPM and hTMSCs, the staining intensity of ALP and ARS increased with the concentration of SPM. In addition, hTMSCs were observed to be bound to the SPM surface, exhibiting directional growth along the surface of elongated SPM. After chemically attaching RITC fluorescent dye to SPM and staining hTMSC F-actin, oriented stretching of hTMSCs along the surface of SPM was observed. As stem cells spread through focal adhesion, YAP/TAZ proteins translocated into the cell nucleus, inducing osteogenesis in stem cells [30,36]. Observing osteogenesis through this mechanotransduction mechanism, an increase in the expression of YAP, TAZ, and CTGF was confirmed from the 3rd day onward. Gelatin-coated TCP was used as a control group. PCR data indicated an increase in osteogenic gene expression, including ALP, OCN, and RUNX2, due to the mechanotransduction induced by SPM in hTMSCs. Through our experiments, we successfully demonstrated all 3 hypotheses proposed for the osteogenic differentiation induction by SPM, suggesting the potential for bone regeneration through similar mechanisms in vivo. Recent studies have revealed that through the intricate interplay between integrin expression and cell–cell adhesion proteins of the cadherin family, mechanical stimuli are converted into biochemical signals, which regulate the organization of the cellular cytoskeleton in the mechanotransduction pathway. It is necessary to further elucidate how the interaction between cells and SPM, as well as the subsequent cell–cell interactions, affect osteogenic differentiation.

Furthermore, in Fig. S5, we compared and evaluated the osteogenic ability of hTMSCs between contact (mixed culture) and noncontact (coculture) conditions. Enhanced ALP and RUNX2 expression by the contact group reveals that SPM induces a strong synergistic effect on osteogenesis through mechanotransduction. Osteogenic gene expression was directly induced by Wnt and ERK signaling pathways due to silica ions, the increased expression of α_V_β_3_ integrin through the interaction between silica particles and MSCs, and, subsequently, the increased expression of downstream pathways such as MAPK, ERK, JNK, and p38, leading to the nuclear translocation of YAP/TAZ, consistent with previous studies and supporting the enhancement of osteogenic gene expression in MSCs during contact with SPM [36–41]. This mechanotransduction pathway mimics a biomimetic environment resembling the process of bone formation in the human body, thus facilitating osteogenesis.

To stably deliver SPM into the body, we integrated it into an enzyme-mediated cross-linkable hydrogel system composed of a mixture of hyaluronic acid and chondroitin sulfate. Hyaluronic acid, known for its high biocompatibility, and chondroitin sulfate, with sulfate groups aiding in biomineralization, formed the basis of this hydrogel [42,43]. Hyaluronic acid and chondroitin sulfate are generally well known for their low cytotoxicity and minimal immunogenicity. They exhibit excellent biocompatibility and form hydrogels through biofriendly reactions. In addition, hyaluronic acid contains amino groups in the glucosamine and d-glucuronic acid units, while chondroitin sulfate includes amino groups in the *N*-acetyl-d-galactosamine and d-glucuronic acid units [44]. These amino acid functional groups are naturally present in the body, contributing to a very mild immunogenic response. Furthermore, although the detailed degradation process has not yet been fully elucidated, spicule is known to exhibit rapid degradation in tissues such as the skin, without showing immunogenicity or tissue toxicity. Spicules are currently being actively researched for their application in dermatological treatments, with their stability proven [14]. Furthermore, our previous research demonstrated that the Ty-mediated hydrogel cross-linking system is rapidly cross-linkable, allowing its use as an injectable type [22,45,46]. The catechol groups generated through the oxidation of tyrosine groups in this system exhibit excellent tissue adhesiveness, promoting integration with endogenous tissue upon in vivo application and potentially enhancing the success rate of transplantation. After introducing tyrosine groups to hyaluronic acid and chondroitin sulfate through EDC/NHS coupling chemistry and enzyme treatment, we confirmed the formation of a porous hydrogel structure. When hTMSCs were cultured in the hydrogel, a high cell survival rate was observed. In addition, despite treatment with hyaluronidase and collagenase following the incorporation of SPM into the hydrogel, low degradability was noted. The mechanical properties and degradation capacity of hydrogel composites are determined by the degree of chemical cross-linking induced by cross-linkers and the physical cross-linking, such as noncovalent bonds between the hydrogel and internal particles. Previous studies have confirmed that enzymes adsorbed on the surface of silica nanoparticles increase the enzyme’s activity and that biopolymers physically adsorb onto the surface of silica nanoparticles [33]. Similarly, in HC_SPM, the degree of cross-linking increased because of this principle, resulting in enhanced physical strength and delayed degradation. These results were also confirmed in degradation experiments involving collagenase treatment. Collagenase can degrade SPM, and as SPM is broken down, the physical cross-linking between the hydrogel and SPM decreases, leading to partial degradation of the hydrogel.

The identified induction of cellular focal adhesion by SPM in vitro intertwined with the activation of the YAP/TAZ pathway, revealing an elaborate regulatory network that promotes enhanced cell adhesion and interaction at the implantation site of bone defects in vivo. The YAP/TAZ pathway, known for its role in mechanotransduction and cell fate determination, appears to act synergistically with SPM-induced focal adhesion to regulate cell behavior and may play a pivotal role in creating a microenvironment osteoconductive to rapid bone regeneration. At the same time, we assume that the observed thicker collagen tissues with increased BV/TV in SPM incorporated hydrogel-implanted site, which is important in the aspect of YAP/TAZ activation. Collagen 1 can act as a downstream target of the YAP/TAZ pathway and contribute to the observed structural strengthening [47,48]. The interdependence between YAP/TAZ-mediated mechanotransduction and collagen enrichment suggests a mechanical cascade that potentially positively amplifies regenerative capacity by providing strong extracellular support for osteoblast activity and mineralized matrix deposition. In addition, the constant release of collagen through SPM degradation is expected to play a crucial role in promoting osteogenic differentiation. Combined with activation of the YAP/TAZ pathway, it can further regulate cell behavior, creating a microenvironment favorable to osteogenesis. Moreover, when considered together with YAP/TAZ activation, the increase in red blood cells observed in histology implies a potential regulatory role for this pathway in the angiogenic process. YAP/TAZ, known to be involved in angiogenesis [48,49], may contribute to the observed heightened vascular response, further enhancing the delivery of oxygen, nutrients, and progenitor cells to the regenerating tissue.

In conclusion, this study investigates the promising osteogenic potential of marine-sponge-derived spicules and collagen-encapsulated silicon dioxide microparticles. This investigation, involving in vitro and in vivo experiments, revealed potential mechanisms by which spicules induce osteogenesis: osteoconduction and mechanotransduction. The in vitro findings demonstrated the role of α_V_β_3_ integrin in successful biomineralization, activation of Wnt and ERK signaling pathways, and promotion of stem cell adhesion to biosilica. Through this mechanistic study, we have confirmed that the osteogenic induction ability of SPM relies more on mechanotransduction via the YAP/TAZ pathway, which mimics the actual bone formation process than on osteoinduction by silica ions. The incorporation of spicules into the hydrogel highlights their bone regenerative potential. The complex interplay between YAP/TAZ pathway activation, collagen enrichment, and angiogenesis presents a comprehensive mechanism to amplify regenerative capacity, providing important insights for the advancement of bone tissue engineering.

### Ethical Approval

All animal experiments in this study were conducted following the guidelines for the Care and Use of Laboratory Animals by the Seoul National University (SNU-230718-8).

## Data Availability

The data that support the findings of this study are available on request from the corresponding author. The data are not publicly available because of privacy or ethical restrictions.
