# Supplementary material for: Biomimetic Marine-Sponge-Derived Spicule-Microparticle-Mediated Biomineralization and YAP/TAZ Pathway for Bone Regeneration In Vivo
Source: Biomater Res. 2024 Jul 25;28:0056. doi: 10.34133/bmr.0056 (PMC11268990; doi:10.34133/bmr.0056)
Supplement: Supplementary 1 — Figs. S1 to S7 Table S1 [file bmr.0056.f1.docx]

**Biomimetic Marine Sponge-Derived Spicules Microparticle-mediated Biomineralization and YAP/TAZ Pathway for Bone Regeneration *in vivo***

*Sumi Choi^a,1^, Jung Hun Kim^b,1^, Tae Hoon Kang^c^, Young-Hyeon An^b,d^, Sang Jin Lee^e^, Nathaniel S. Hwang^b,c,d,*^, Su-Hwan Kim^a,^**

^a^ Department of Chemical Engineering (BK21 FOUR), Dong-A University, Busan, 49315, Republic of Korea

^b^ School of Chemical and Biological Engineering, Seoul National University, Seoul, 08826, Republic of Korea

^c^ Interdisciplinary Program in Bioengineering, Seoul National University, Seoul 08826, Republic of Korea

^d^ Bio-MAX/N-Bio, Seoul National University, Seoul, 08826, Republic of Korea

^e^ Biofunctional Materials, Division of Applied Oral Sciences and Community Dental Care, Faculty of Dentistry, The University of Hong Kong, 34 Hospital Road, Sai Ying Pun, Hong Kong Special Administrative Region

^1^ S. Choi and J.H. Kim contributed equally to this work.

*To whom correspondence should be addressed.S.H. Kim ([suhwankim@dau.ac.kr](mailto:suhwankim@dau.ac.kr)) or N.S. Hwang (nshwang@snu.ac.kr)

**
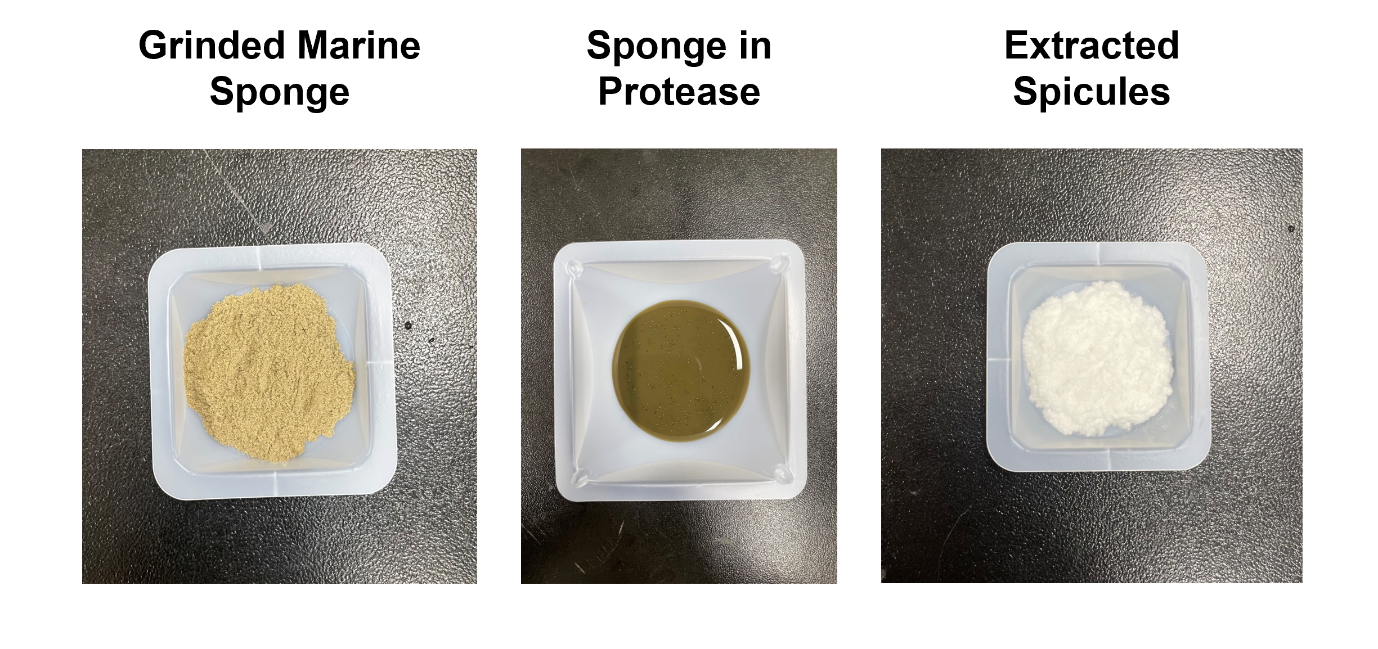
**

**Supplementary Figure 1.** Images of procedures of spicule extraction from marine sponges.


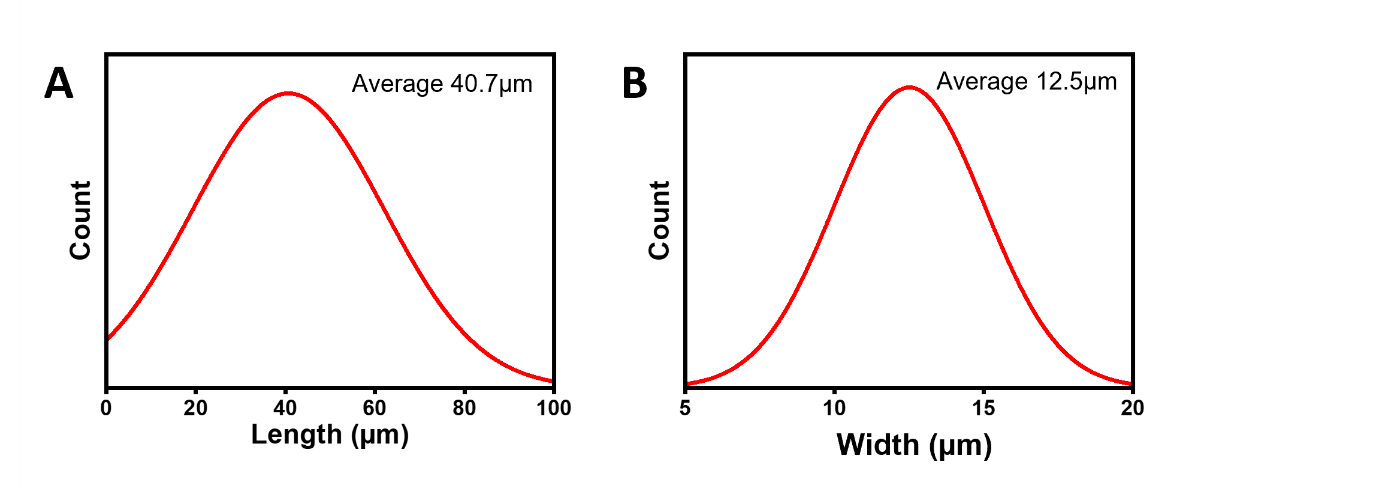


**Supplementary Figure 2.** SPM size (A) Length; average 40.7 μm, (B) Width: average 12.5 μm


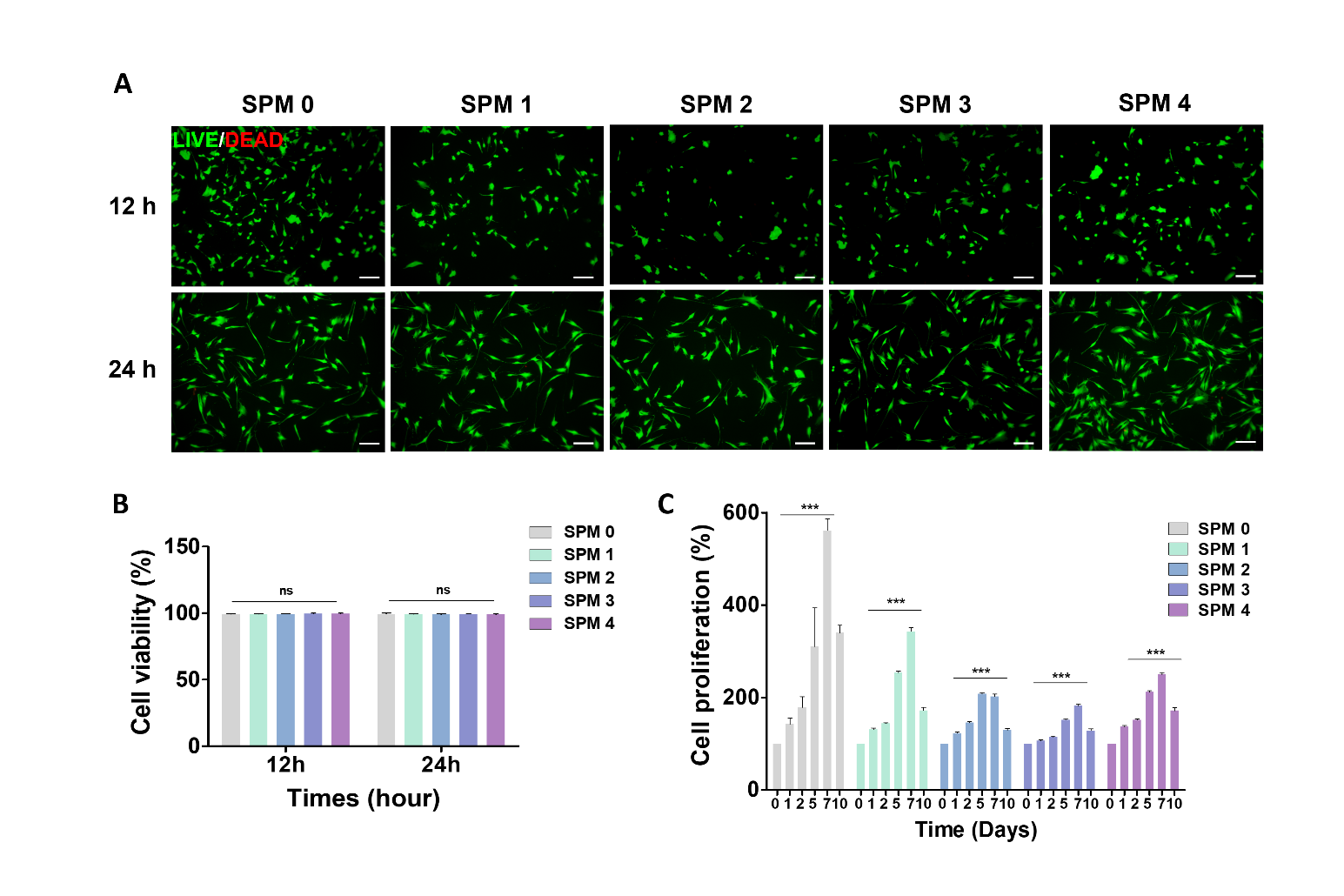


**Supplementary Figure 3.** (A) Live/Dead images of hTMSCs cultured with SPM; scale bar=50 μm. (B) Quantification of hTMSCs Live/Dead assay. (C) Quantification of hTMSCs proliferation. Data are presented as mean ± SD (n = 6). (ns > 0.05, *p < 0.05, **p < 0.01, ***p < 0.001)


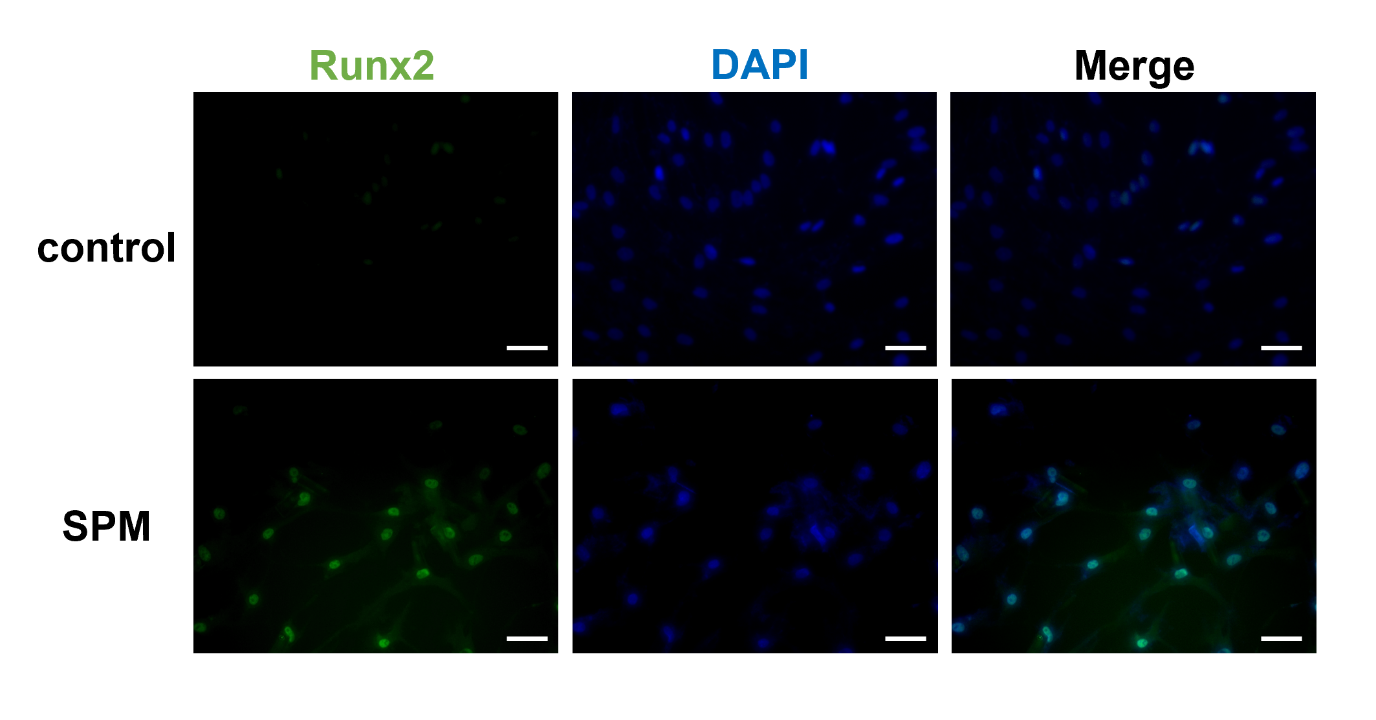


**Supplementary Figure 4.** Immunocytochemistry (ICC) staining of osteogenic factors (Runx2) in hTMSCs cultured with and without SPM for 7 days; green (RunX2), blue (nucleus), scale bar=50 μm.


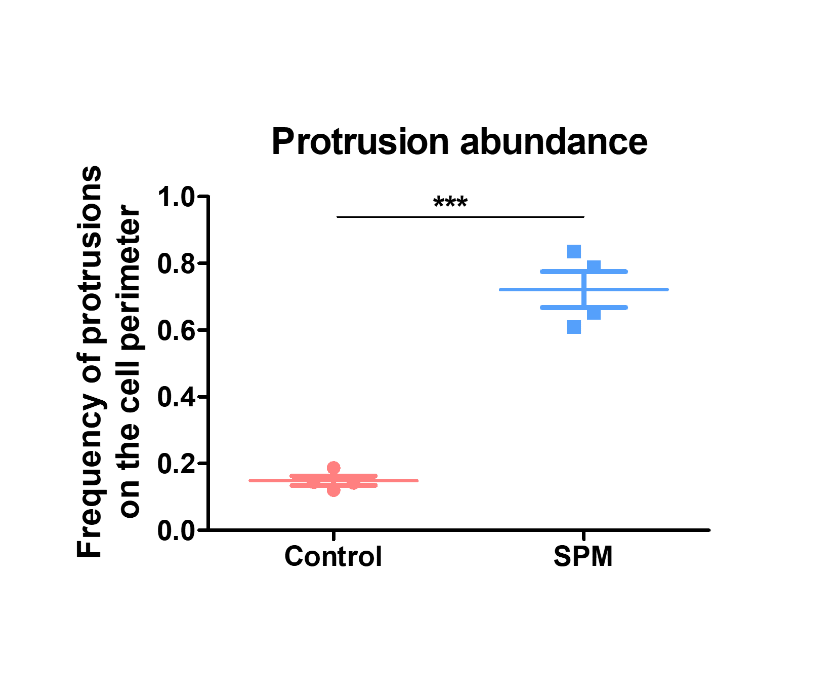


**Supplementary Figure 5.** The number of protrusions per 10μm length of the hTMSCs perimeter; Control: without SPM


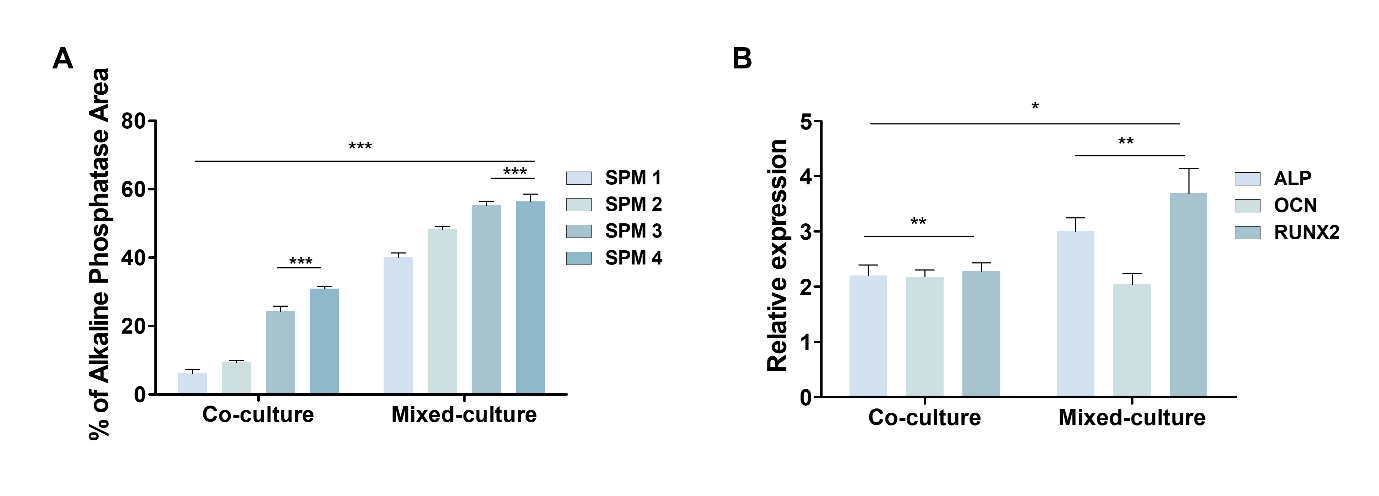


**Supplementary Figure 6. (A)** Quantification of ALP deposition areas in hTMSCs co-cultured and mixed cultured with SPM for 7 days, as determined by ALP staining. (B) Comparison of osteogenic gene expression (ALP, RUNX2, OCN) in hTMSCs co-cultured and mixed cultured with SPM for 7 days. Data are presented as mean ± SD (n = 6). (ns > 0.05, *p < 0.05, **p < 0.01, ***p < 0.001)


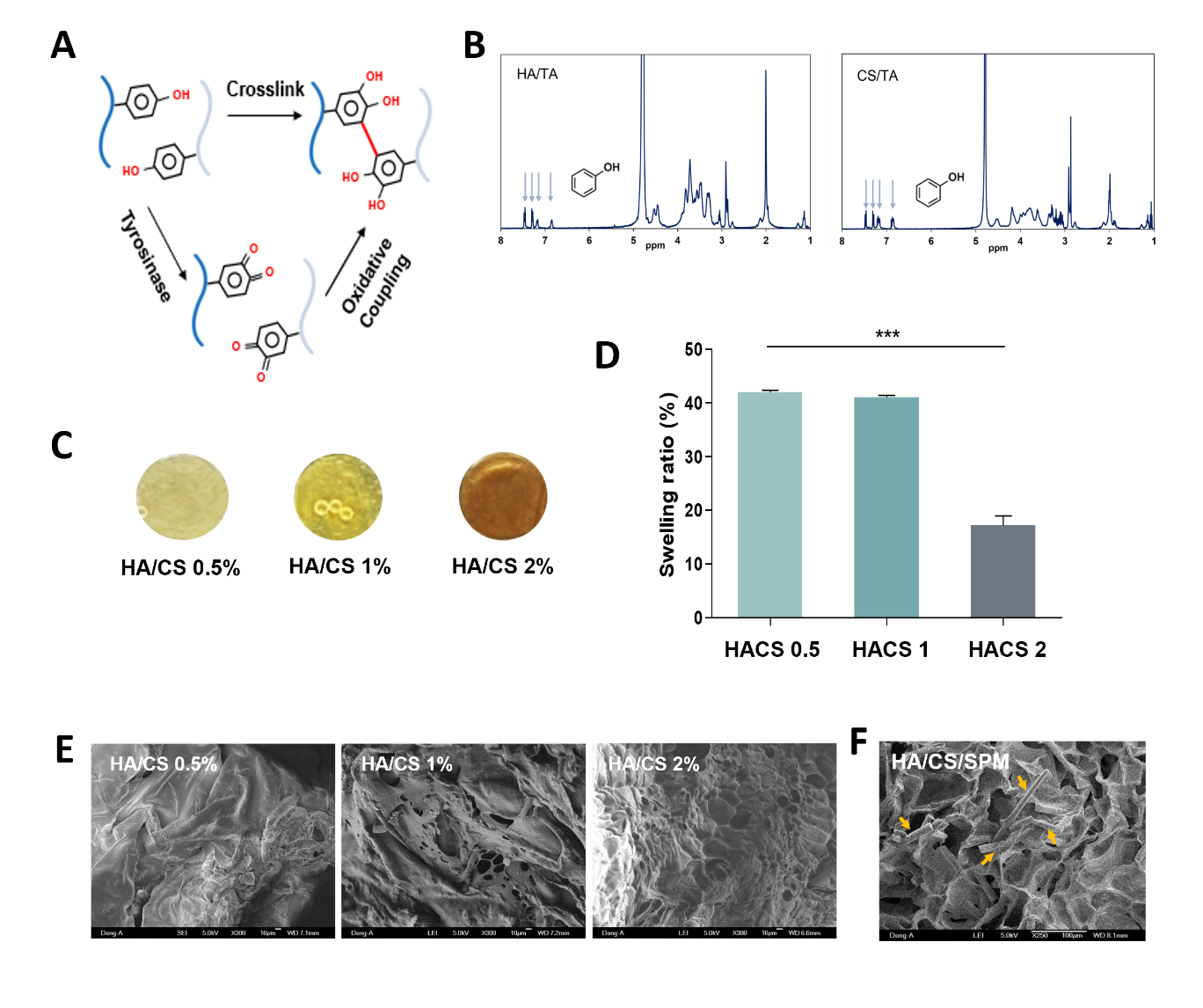


**Supplementary Figure 7.** (A) Schematic illustration of the mechanism of Ty-mediated hydrogel crosslinking. (B) NMR data of tyramine conjugated hyaluronic acid and chondroitin sulfate. (C) Images of hydrogels. (D) Analysis of hydrogel swelling rate after incubation in PBS for 24 hours. (E) SEM images of each hydrogel. (F) SEM image of HC_SPM. Data are presented as mean ± SD (n = 3). (ns > 0.05, *p < 0.05, **p < 0.01)

]

| **gene** | **primer 5′-3′** |
| --- | --- |
| *GAPDH* | F: CGC TCT CTG CTC CTC CTG TT |
|  | R: CCA TGG TGT CTG AGC GAT GT |
| *RunX2* | F: ACT GGG CCC TTT TTC AGA |
|  | R: GCG GAA GCA TTC TGG AA |
| *OCN* | F: GCC TTT GTG TCC AAG C |
|  | R: GGA CCC CAC ATC CAT AG |
| *ALP* | F: ACG TGG CTA AGA ATG TCA TC |
|  | R: CTG GTA GGC GAT GTC CTT A |
| *COL1* | F: GTC ACC CAC CGA CCA AGA AAC C |
|  | R: AAG TCC AGG CTG TCC AGG GAT G |
| *YAP* | F: CAA CTC CAA CCA GCA GCA AC |
|  | R: TTG GTA ACT GGC TAC GCA GG |
| *TAZ* | F: TGG ACC AAG TAC ATG AAC CAC C |
|  | R: TGC CTT CTA TGC TCC CTC CT |
| *CTGF* | F: AGG AGT GGG TGT GTG ACG A |
|  | R: CCA GGC AGT TGG CTC TAA TC |

**Supplementary Table 1.** Primer lists related to osteogenesis and mechanotransduction.
